# Understanding the chemistry of the artificial electron acceptors PES, PMS, DCPIP and Wurster’s Blue in methanol dehydrogenase assays

**DOI:** 10.1007/s00775-020-01752-9

**Published:** 2020-02-14

**Authors:** Bérénice Jahn, Niko S. W. Jonasson, Hurina Hu, Helena Singer, Arjan Pol, Nathan M. Good, Huub J. M. Op den Camp, N. Cecilia Martinez-Gomez, Lena J. Daumann

**Affiliations:** 1 Department of Chemistry, Ludwig-Maximilians-Universität München, Butenandtstr. 5-13, 81377 Munich, Germany; 2 Department of Microbiology, Institute of Wetland and Water Research, Radboud University, Nijmegen, The Netherlands; 3 Department of Microbiology and Molecular Genetics, Michigan State University, East Lansing, MI USA

**Keywords:** Methanol dehydrogenase, Enzymatic assay, Coupled assay, UV–Vis spectroscopy, EPR spectroscopy, Electron acceptors, PMS, PES, Wurster’s blue, DCPIP

## Abstract

**Abstract:**

Methanol dehydrogenases (MDH) have recently taken the spotlight with the discovery that a large portion of these enzymes in nature utilize lanthanides in their active sites. The kinetic parameters of these enzymes are determined with a spectrophotometric assay first described by Anthony and Zatman 55 years ago. This artificial assay uses alkylated phenazines, such as phenazine ethosulfate (PES) or phenazine methosulfate (PMS), as primary electron acceptors (EAs) and the electron transfer is further coupled to a dye. However, many groups have reported problems concerning the bleaching of the assay mixture in the absence of MDH and the reproducibility of those assays. Hence, the comparison of kinetic data among MDH enzymes of different species is often cumbersome. Using mass spectrometry, UV–Vis and electron paramagnetic resonance (EPR) spectroscopy, we show that the side reactions of the assay mixture are mainly due to the degradation of assay components. Light-induced demethylation (yielding formaldehyde and phenazine in the case of PMS) or oxidation of PES or PMS as well as a reaction with assay components (ammonia, cyanide) can occur. We suggest here a protocol to avoid these side reactions. Further, we describe a modified synthesis protocol for obtaining the alternative electron acceptor, Wurster’s blue (WB), which serves both as EA and dye. The investigation of two lanthanide-dependent methanol dehydrogenases from *Methylorubrum extorquens* AM1 and *Methylacidiphilum fumariolicum* SolV with WB, along with handling recommendations, is presented.

**Graphic abstract:**

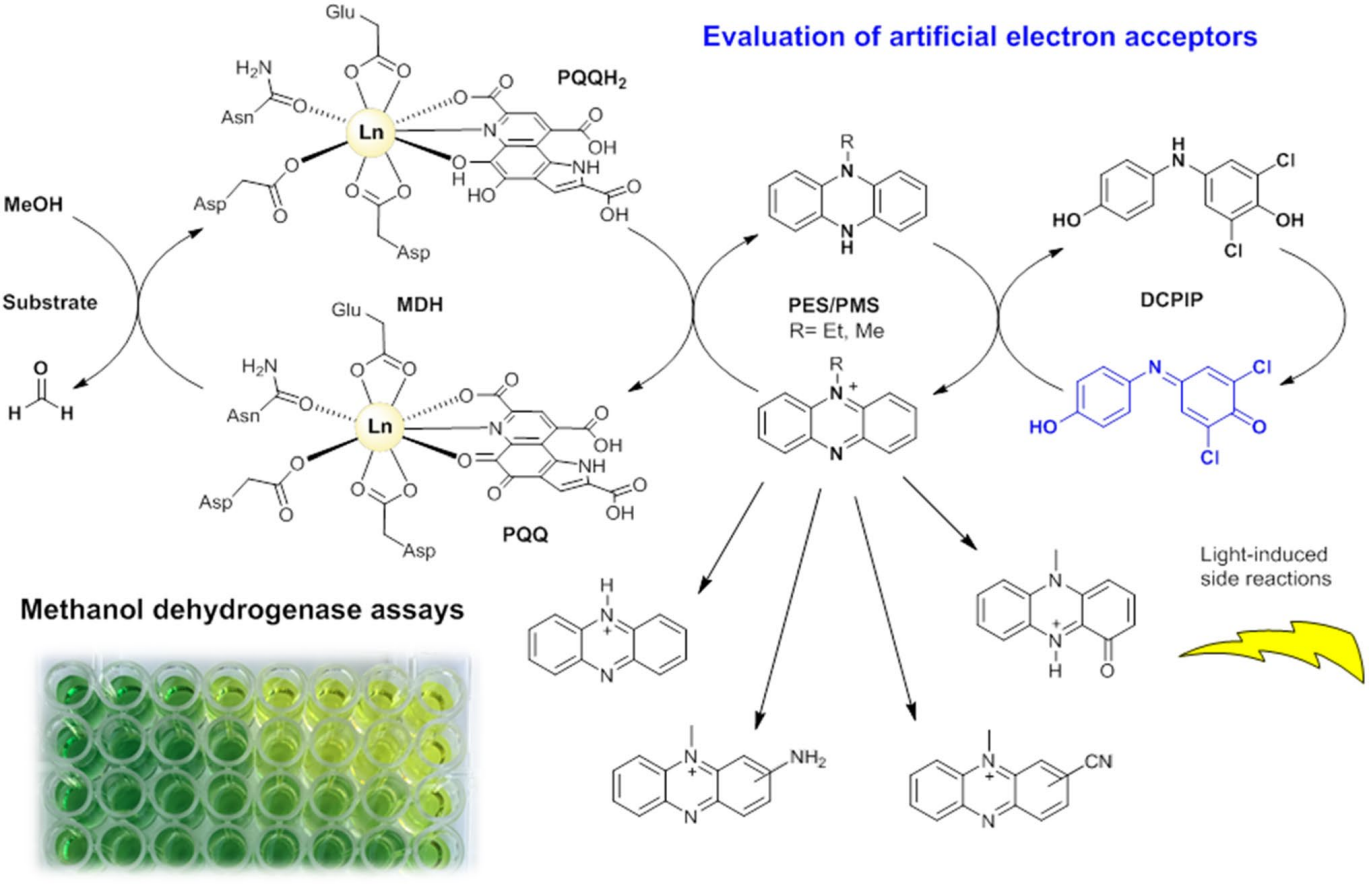
 Lanthanide-dependent methanol dehydrogenases. Understanding the chemistry of artificial electron acceptors and redox dyes can yield more reproducible results.

**Electronic supplementary material:**

The online version of this article (10.1007/s00775-020-01752-9) contains supplementary material, which is available to authorized users.

## Introduction

**Chart 1:**
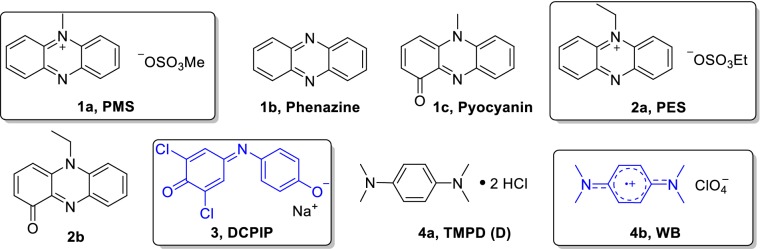
Electron acceptors and dyes that have been used to assess MDH activity (their degradation products are also shown): PMS (**1a**), phenazine (**1b**) and its oxidation product pyocyanin (PMS_ox_, **1c**). PES, (**2a**) and its oxidation product (PES_ox_, **2b**). DCPIP, (**3**), *N*,*N*,*N*′,*N*′-tetramethyl-p-phenylenediamine (TMPD) dihydrochloride (TMPDD, **4a**) and *N*,*N*,*N*′,*N*′-tetramethyl-*p*-phenylenediamine perchlorate (Wurster’s blue, WB, **4b**)

Biochemical assays are powerful analytical techniques used to identify or quantify proteins, to study the binding of substrates and inhibitors, and to measure the activity of enzymes. The family of methanol dehydrogenase enzymes (MDH) has recently taken the spotlight again after it was discovered that many bacteria utilize lanthanide-dependent MDH of the XoxF family [1–7]. This finding has fueled an entirely new area of research—lanthanide-dependent bacterial metabolism and biochemistry. The activity of methanol dehydrogenases in vitro is routinely measured using the convenient spectrophotometric method developed by Anthony and Zatman [8]. The electron transfer from the substrate, either methanol or formaldehyde, via the redox cofactor pyrroloquinoline quinone (PQQ) in the active site is coupled to electron acceptors (EA). Because of the absence of visible light-absorbing substrates or products, a dye is required for the read-out of the assay. Usually, artificial electron acceptors, such as phenazine methosulfate (PMS), phenazine ethosulfate (PES), *N*,*N*,*N*′,*N*′-tetramethyl-*p*-phenylenediamine (TMPD) derivatives like its radical cation (Wurster’s blue, WB) or 2,6-dichlorophenolindophenol (DCPIP), are involved. The first two are the most widely used EA and the latter two serve as redox dyes (Chart 1) [8–10]. The electron transfer in MDH enzymes in vivo is proposed to take place in distinct one-electron steps [11, 12]. PMS, PES and WB enable the regeneration of the prosthetic group pyrroloquinoline quinone by mimicking cytochrome *c*_L_ or cytochrome *c*_GJ_, the physiological electron acceptors of these enzymes [9, 13–15]. Besides colorimetric techniques, an amperometric approach has been used to assess MDH activity. Here, methanol conversion is coupled to electron acceptors that are, in turn, linked to oxygen in an oxygen-sensitive electrode [16, 17]. Studies with the natural electron acceptor cytochrome *c*_L_ and a bovine or equine heart cytochrome as terminal electron acceptor and dye have also been reported [15, 16, 18]. Recently, the oxidation of methanol by Eu-MDH via cytochrome *c*_GJ_ driven by electrocatalytic voltammetry was also demonstrated [14].

While the implementation of the colorimetric assay for the analysis of MDH activity is facile, many difficulties regarding the reproducibility of assay results have been reported [19, 20]. In light of the importance of MDH assays for the recently established field of lanthanide biochemistry, we revisit this assay and its components from a chemist’s point of view. We provide explanations and solutions to avoid side reactions occurring in the assay mixture under different conditions. We are convinced that it is important to understand the underlying chemistry and side reactions of the artificial electron acceptors to avoid fluctuations in composition and concentration of the assay mixture, ultimately yielding more reproducible assay results.

## Results and discussion

### A note on MDH

MDH activity is often observed in the absence of an added substrate [9]. We stress here that the investigation of assay components PES/PMS and DCPIP does not solve the problem with this so-called endogenous substrate of MDH, but shall identify handling errors while performing colorimetric assays. It has been suggested that the endogenous substrate could stem from traces of alcohol left from the recrystallization of the buffer. An inquiry with the supplier ruled this out, as no alcohol had been used during the final purification stages of our buffer (PIPES). However, an experiment with NaCl (concentrations between 10 and 100 mM were tested) showed hardly any background reaction in the absence of the substrate compared to the PIPES buffer (10 and 100 mM tested). This background reaction from endogenous substrate can also be observed in PIPES buffer when MDH is assayed with its natural electron acceptor (e.g., cytochrome *c*_GJ_). Hence, traces of other organic substances in the buffer that could act as substrates cannot be ruled out. Further, we and others have observed significant variations of MDH activity among different enzyme batches and fractions obtained after purification. Fractions exhibiting lower enzymatic activity often show a decreased PQQ absorbance (as observed around 355 nm) relative to the 280 nm feature or the complete loss of the prosthetic group (data not shown) [21]. Since the proteins often have to be stored in methanol for stability, washing of MDH before conducting assays is required. Due to this procedure, a partial removal of PQQ in the active site is conceivable. Hence, full spectra (from 200 to 600 nm) should always be recorded to include the PQQ fingerprint (the absorbance spectra of the used MDH samples are presented in Figure S13), and, in addition to SDS-PAGE, 355/280 ratios should be reported to normalize for the holoenzyme content of the sample [22, 23].

### The redox dye DCPIP

**Scheme 1:**
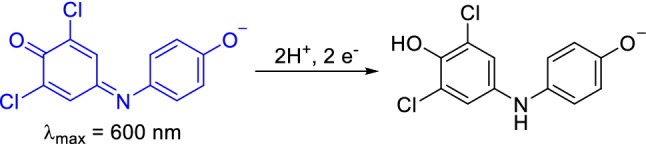
Upon two electron reduction, DCPIP undergoes a distinct color change. Usually the sodium salt and neutral to alkaline pH are employed in MDH assays. Hence, one of the deprotonated forms is shown

**Fig. 1:**
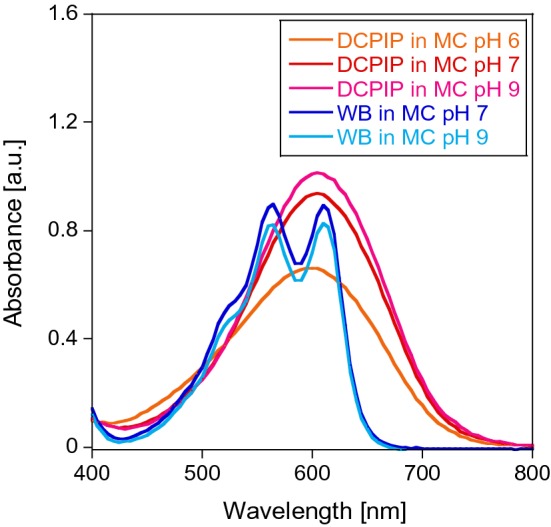
Absorbance spectra of 50 µM DCPIP (**3**) and 100 µM WB (**4b**) in 100 mM multicomponent buffer of pH 6 (for DCPIP), pH 7 or pH 9. Fresh samples were prepared by diluting a 2 mM stock solution of the dye with the corresponding buffer. Spectra were collected at a Cary60 UV–Vis spectrophotometer at room temperature and corrected for the buffer baseline

DCPIP has been used for decades as a redox dye and two-electron acceptor [24–26]. A wavelength of 600 nm is routinely used for the detection of DCPIP-coupled reactions (Scheme 1) mostly for assessing MDH activity together with PMS (**1a**) and PES (**2a**), although studies of coupling DCPIP with the natural electron acceptor cytochrome *c*_L_ have been reported [27]. The comparability of results relies often on the reported extinction coefficient *ε* at 600 nm. However, vastly varying values for ε_600_ have been published even for similar conditions (Table 1). ε_600_ of DCPIP is pH dependent (Fig. 1) and increases with increase in pH (this dye has a pK_a_ around 5.90) [28]. Furthermore, a redox potential of + 217 mV has been reported [29].

**Table 1:** Extinction coefficients of DCPIP in different buffer systems, at different pH values and temperatures that have been reported in the literature

Extinction coefficient ε_600nm_ (mM^−1^ cm^−1^)	Buffer system	pH	Temperature (°C)
14.0 [28]	Phosphate	6.05	26
6.6 [63]	–	6.05	–
18.5 [64]	Phosphate	6.50	21
20.6 [28]	Phosphate	7	26
19.1 [65-67]	Phosphate	7	–
21.0 [64]	Phosphate	7	20–30
17.8 [64]	Phosphate	7	20
16.1 [68]	Phosphate	7	30
18.5 [19]	PIPES	7.2	45
19.1	Tris–HCl	8	30
21.5 [60]	–	8	–
21.8 [28]	–	8	26
21.9 [28]	–	8.3	26
21.9 [20]	Tris–HCl	8.5/9	26
19.0 [69]	Tris–HCl	9	30
21.0 [46, 70]	Tris–HCl	9	30
21.5 [60]	Tris–HCl	8	-
21.9 [20]	Tris–HCl	9	26
22.0 [48]	CHES	9	30
7.8 ± 0.2	Multicomponent buffer^a^	5.3^b^	45
11.3 ± 0.3	Multicomponent buffer^a^	5.7^b^	45
14.4 ± 0.5	Multicomponent buffer^a^	6.4^b^	45
17.9 ± 0.5	Multicomponent buffer^a^	6.7^b^	45
18.8 ± 0.5	Multicomponent buffer^a^	7.1^b^	45
19.7 ± 0.5	Multicomponent buffer^a^	7.4^b^	45
19.7 ± 0.4	Multicomponent buffer^a^	8.7^b^	45

^a^Multicomponent buffer: 2.5 mM citric acid, 2.5 mM Bis–Tris, 2.5 mM Tris and 2.5 mM CHES [14]

^b^Corrected pH of buffer at 45 °C [35]

Variations of the reported extinction coefficient, even for similar conditions, cannot be solely attributed to different batches and purities of the DCPIP dye used (Table 1). MDH assays are run at different pH values and temperatures depending on the MDH source (extremophile, mesophile, acidophile, neutrophile, etc.). Therefore, it is important to determine the extinction coefficient of DCPIP for new assay conditions (buffer system, pH, temperature). Our measurements further showed that the solubility of DCPIP has likely been overestimated. A concentration of 10 mg DCPIP/ml water is described to be the solubility limit.

However, we found 2 mM of DCPIP (0.65 mg/ml) to be a good concentration in MilliQ water to give a homogeneous solution without precipitate. Whereas the powdered form of the dye is reported to be stable, DCPIP solutions should be prepared freshly every day in dark reaction tubes, as a low color stability of DCPIP in solution has been described [30, 31]. Interestingly, we observed that in DCPIP-coupled MDH assays, the enzymatic activity was higher under exclusion of oxygen compared to assays performed under aerobic conditions. This is most likely due to the slow re-oxidation of reduced DCPIP under aerobic conditions [32]. Also the bleaching of the dye in the absence of MDH was significantly decreased when oxygen was absent (data not shown). Therefore, only freshly filtered (and thus somewhat degassed) buffers should be used.

### A note on buffers

**Chart 2:**
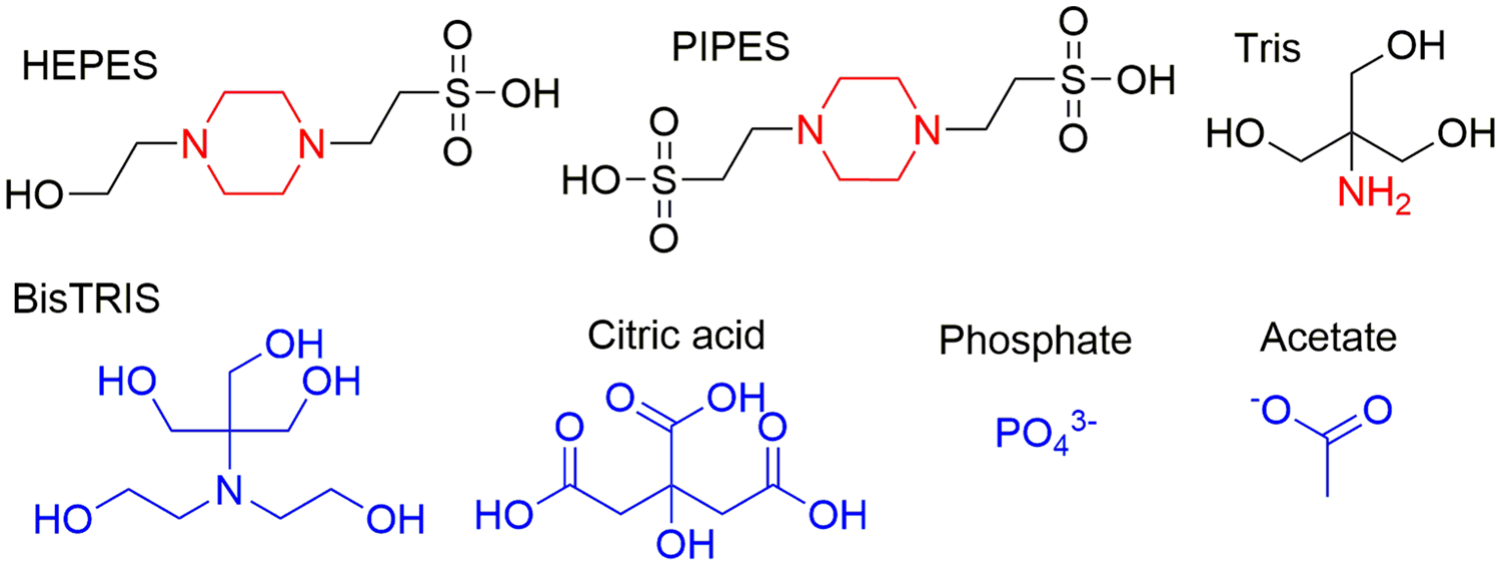
A selection of buffers that have been used in MDH assays. The piperazine ring in PIPES and HEPES shown in red may cause problems when investigating redox reactions. The amine of Tris can react with formaldehyde, a substrate/product of many enzymes including MDH. Buffers shown in blue are known to complex or precipitate lanthanides and may thus compete with the enzyme for the metal ion in the active site

Many bacteria that express methanol dehydrogenases grow best at elevated temperatures. Examples are the genera *Methylothermus*, *Methylococcus*, *Methylocaldum* and *Methylacidiphilum *(e.g., *M. fumariolicum* SolV or *M. infernorum* V4) [33, 34]. Hence, the assay of the isolated enzyme is often conducted at temperatures other than room temperature. As many buffers exhibit a change in pH upon heating, it is important to account for the concomitant change in pH as well [35]. It is thus advisable to either correct the pH at a certain temperature or to determine ε_600_ of DCPIP for the given conditions (type of buffer, pH, temperature) to ensure better comparability between assays. Furthermore, Grady, Chasteen and Harris report that 4-(2-hydroxyethyl)-1-piperazineethanesulfonic acid (HEPES) and piperazine-*N*,*N*′-bis(2-ethanesulfonic acid) (PIPES) and other piperazine-based buffers readily show radical formation (Chart 2) [36]. This is especially troublesome when studying redox reactions. Phosphate ions are known to readily precipitate supplemented lanthanides [37]. Tris(hydroxymethyl)-aminomethane buffer (Tris) is strongly temperature dependent and can further undergo Schiff base-type condensations with aldehydes, which is problematic when investigating substrates like formaldehyde [38, 39]. Additionally, the Tris buffer family shows complex formation with many metal ions as well as succinate and some members of the cyclohexylamino, acetamido and propanol family of buffers [40]. A complexation of lanthanides was further described for citrate and Good’s buffers such as tricine, which will disturb metal-binding studies [41]. While there may not exist a perfect buffer system, it is important to be aware of the aforementioned potential pitfalls (Chart 2).

### Artificial electron acceptors PES and PMS

Phenazines are applied as primary EA in DCPIP-coupled assays, replacing the physiological electron acceptor, cytochrome *c*_L_ or cytochrome *c*_GJ_ in artificial assays [14, 15]. Although both phenazines are widely used as electron acceptors and Ghosh and Quayle reported PES as the preferred electron acceptor [42], PMS is predominantly utilized in MDH assays [20, 43, 44]. In the chemistry community it is well known that PMS shows a higher tendency for radical formation, dealkylation and decomposition than PES [42, 45]. However, few of these insights have made their way into the life science field. Hence, to better understand the stability and handling of these electron acceptors in a biochemistry setting and to prevent a decrease of the phenazine concentration, we investigated them more closely.

1. Stability of PMS and PES under storage and assay conditions

**Fig. 2:**
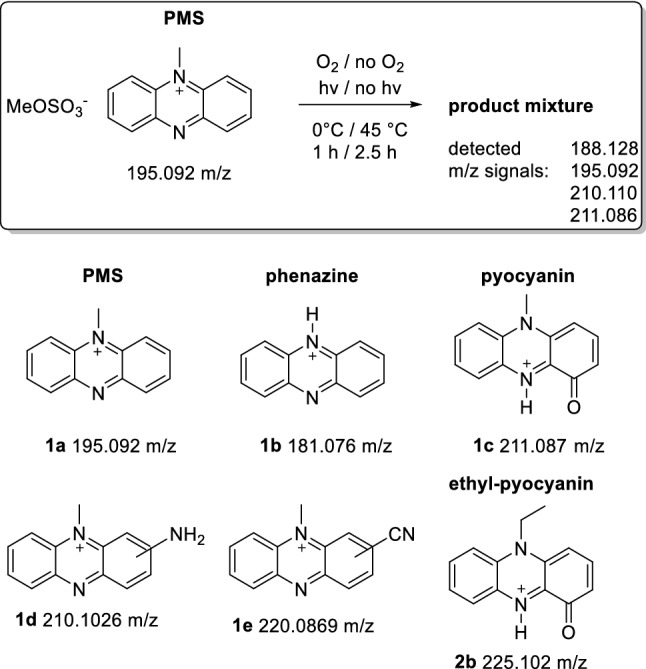
PMS was exposed to different conditions and the product mixture was analyzed using mass spectrometry. Structures and exact masses of the cations of PMS, phenazine (as its protonated derivative), and pyocyanin (as its protonated derivative). Products of the reaction of PMS with ammonia and cyanide, according to the literature [61, 62], and a proposed structure of ethyl-pyocyanin, a decomposition product of PES, are also shown (for more details see Supporting Information)

The stability of PES and PMS toward light, oxygen, temperature, pH and nucleophiles was investigated with mass spectrometry (MS) and EPR spectroscopy to shed light onto side reactions that may occur under storage and MDH assay conditions. High-resolution (HR) MS showed that PMS does, indeed, decompose when exposed to light, especially at elevated temperatures. Also, the presence of oxygen seems to determine the outcome of the decomposition reaction. Pyocyanin (211.087 *m/z*) has been identified as a possible decomposition product (see Fig. 2), but did not act as an artificial electron acceptor itself (data not shown).

Further, when cyanide and/or ammonia was added, phenazine (181.076 *m/z*) was identified as a decomposition product (for more details see Supporting Information). In addition to a decrease in concentration of the electron acceptor, this demethylation also leads to the formation of formaldehyde as by-product which is troublesome as this can serve as a substrate for the investigated enzyme system. PES showed similar behavior, although decomposition to phenazine was observed only in miniscule amounts. Additionally, we measured the mass spectrum of a complete assay mixture containing 1 mM PES, 100 µM DCPIP and 20 µM EuCl_3_ in 20 mM PIPES buffer and observed only minor amounts of decomposition products, confirming that the exclusion of light was enough to reduce the decomposition of the assay mixture.

**Fig. 3:**
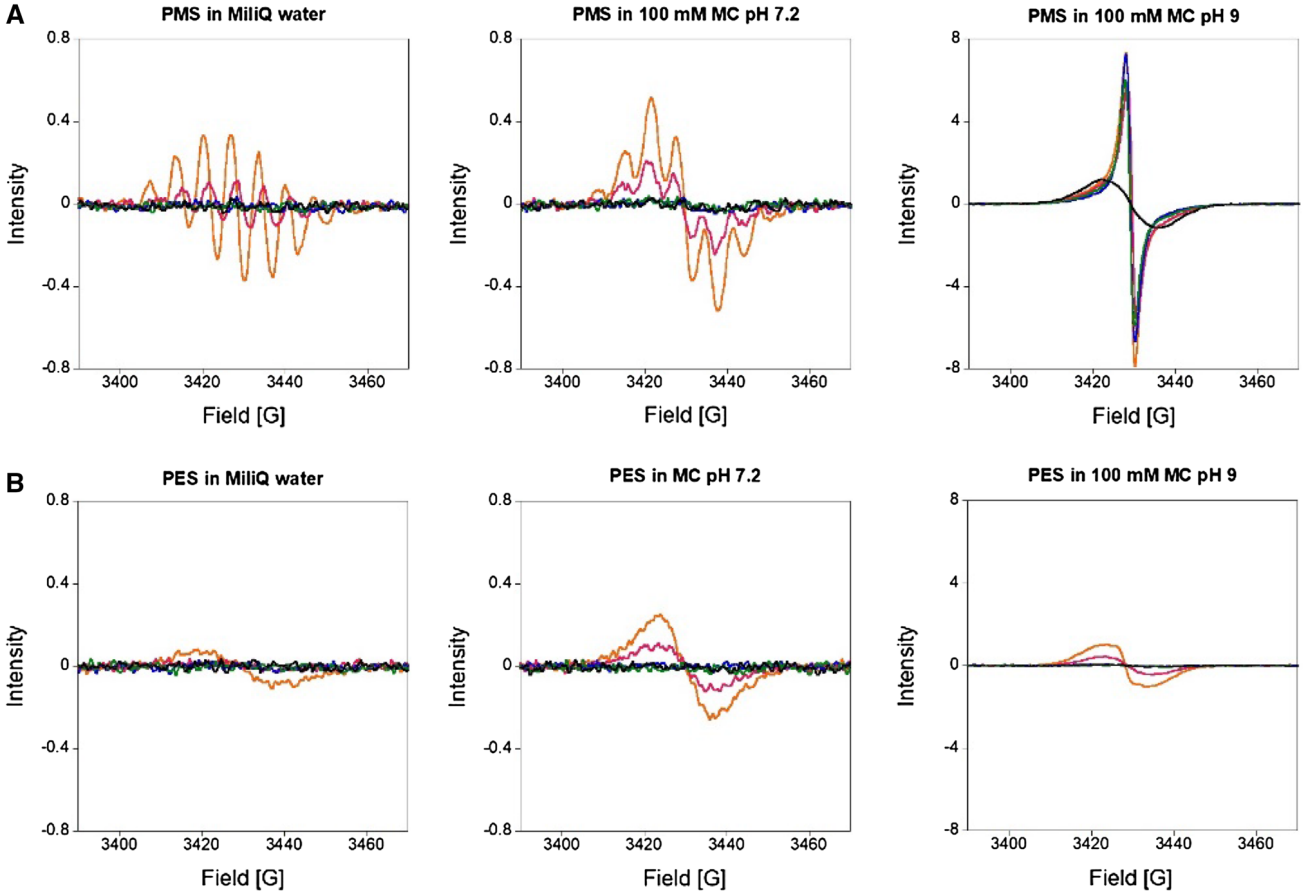
EPR spectra of 10 mM PMS (**a**) and PES (**b**) in MilliQ water (pH 6) or 100 mM multicomponent (MC) buffer pH 7.2 or pH 9. Solutions were prepared on a cloudy day and were either stored in an amber tube at 4 °C (black line), at RT in the dark (green line) or heated at 45 °C for 15 min in an amber tube (blue line). Additional samples were exposed to either daylight (orange line) or UV light of 254 nm (pink line) for 5 min each. Spectra were recorded at room temperature using an EMXnano EPR spectrometer

**Fig. 4:**
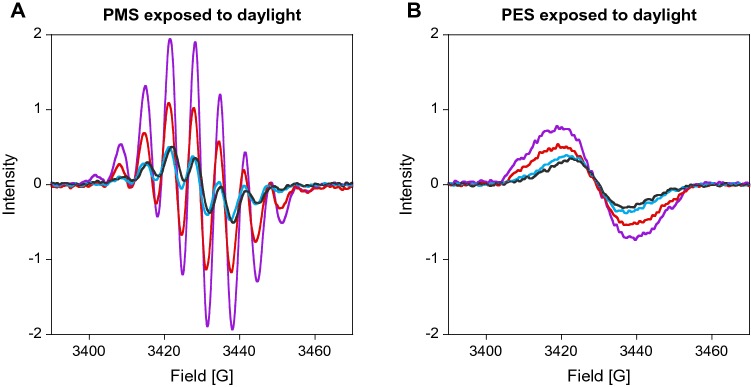
EPR spectra of 10 mM PMS (**a**) and PES (**b**) in MilliQ water (pH 6, purple line); 20 mM PIPES buffer of pH 6.2 (red line) and pH 7.2 (blue line); 20 mM potassium phosphate buffer of pH 7.2 (grey line). Solutions were prepared on a sunny day and were exposed to daylight for 5 min. Spectra were recorded at RT using an EMXnano EPR spectrometer

To investigate whether light-induced degradation proceeds via radical formation under certain conditions (light, pH, temperature), EPR spectroscopy was used (Figs. 3, 4). First, PMS and PES were analyzed in MilliQ water and buffered aqueous solution at pH 7.2 and 9, the same conditions that we used in dye-coupled assays (100 mM multicomponent (MC) buffer, Fig. 3).

We observed more rapid radical formation for PMS than for PES and, in both samples, the level of formed radicals was increased at alkaline pH (pH 9 resulting in a 16 × higher EPR intensity) when the samples were exposed to daylight. UV light (254 nm) led to a similar, but much smaller effect. Heating the solutions of electron acceptors prior to use, as has been recommended [46], led to little radical formation, and neither did storage of the aqueous stock solutions at 4 °C. These results are in line with those of the literature [42, 47]. Our results also indicate that radical formation is influenced not only by light exposure (sample preparation on sunny and cloudy days already showed a different radical content), but also by the pH and the buffer system (Fig. 4).

To sum up, it is recommended using PES instead of PMS and to diligently prevent light exposure. Stock solutions of these artificial EA should be prepared fresh in MilliQ water instead of buffer and the assay mixture should be heated subsequently for at least 15 min prior to performing experiments. It is further advisable to study the absorbance of the assay mix over time in the absence of MDH upon switching to a new buffer system.

2. Difference between using PES or PMS and different batches of these electron acceptors in an MDH assay

**Fig. 5:**
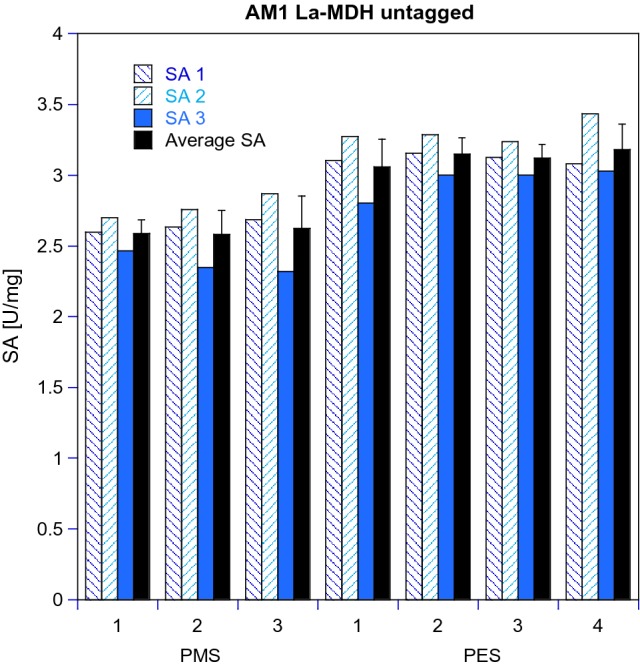
Specific activity (SA, in μmol min^−1^ mg^−1^) of MDH using different PMS and PES batches of different purities and suppliers. *M. extorquens* AM1 La-MDH (untagged, 100 nM) in multicomponent buffer (100 mM, pH 9), 15 mM NH_4_Cl at 30 °C. All samples contained 100 μM DCPIP and 50 mM MeOH, with 1 mM PES or PMS. Total volume in all wells was 200 μL. The reaction was monitored at 600 nm. SA1 and SA3 were determined by a different pair of hands than SA2 and are technical replicates

During our studies, we noted differences both in the appearance and spectroscopic signatures of commercial PMS and PES samples. Table S8 shows that the elemental composition of the samples varies only within the error of the used instrument (0.30%) for both phenazine derivatives obtained from Sigma-Aldrich®, whereas the PMS sample obtained from abcr® shows a significantly lower carbon content. This sample also showed different IR and UV/Vis spectra compared to the PMS samples from Sigma-Aldrich® (see Supporting Information Figures S2–S3 for more details). However, when used to determine the activity of MDH enzymes (originated from both strains AM1 and SolV), the three PMS samples yielded similar results (See Fig. 5 for AM1, data obtained for SolV MDH not shown). It was observed that PES gave higher specific activities for both AM1 and SolV MDH, and that the shelf life or LOT# of the EA did not influence the assay (Table S1).

### A combined one-electron acceptor and redox dye in one: Wurster’s blue

**Scheme 2:**
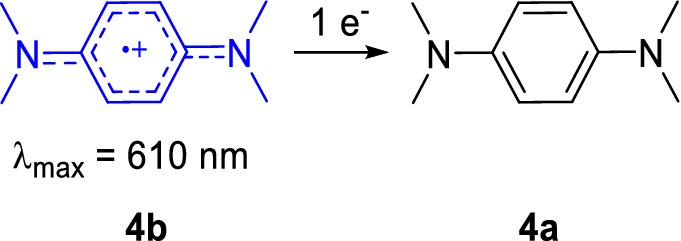
The radical cation Wurster’s blue (**4b**) can undergo reduction to TMPD (**4a**) and can be used to monitor MDH activity

Besides the two-component assay system with the two-electron acceptors PMS/PES and DCPIP, the one-electron acceptor and radical cation Wurster’s blue (WB, **4b** in Scheme 2) can be used for the investigation of methanol dehydrogenases [9]. We refer herein to the cation radical of TMPD (**4a**) as WB. WB has been used for respiration studies in biochemistry and, many decades ago, also as an electron acceptor for alcohol dehydrogenases [9, 12, 48, 49]. The absorption spectrum of a 100 µM WB solution is shown in Fig. 1. From a chemical point of view, the properties of WB and its precursor, TMPD, have been extensively studied in the past [50–55], but their characteristics and handling conditions are not commonly known in the life science field. Therefore, we synthesized WB using a modified protocol (Supporting Information) according to Michaelis and Granick from the commercially available TMPDD and analyzed WB under different storage as well as MDH assay conditions to optimize its use in biochemical assays [56]. We found that **4b** is fairly stable as a solid for several weeks at room temperature under an atmosphere of nitrogen. Storage under an atmosphere of nitrogen at − 20 °C, however, is recommended for better stability.

Previously reported extinction coefficients of WB are presented in Table 2. Additionally, we determined ε_610nm_ at different pH values in a multicomponent buffer under the same conditions as we used in MDH assays. As shown in Table 2 and Fig. 1, the extinction coefficient varies to a lesser extent compared to DCPIP.

1. Storage conditions

**Fig. 6:**
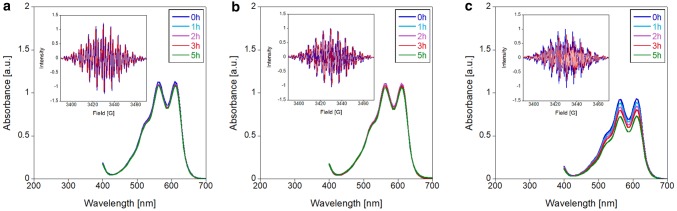
UV–Vis and EPR spectra of 200 µM WB in solution over time. 2 mM WB samples in MilliQ water (**a**, **b**) or 100 mM multicomponent buffer pH 9 (**c**) were stored on ice. In the case of **a** WB was diluted with MilliQ water. Samples of **b** and **c** were diluted in 100 mM multicomponent buffer, pH 9. UV–Vis spectra of triplicates (**a**, **c**) and duplicates (**b**) were recorded at 30 °C on an Epoch2 spectrophotometer without path length correction. MilliQ water and buffer baselines were subtracted from the corresponding spectra. The standard deviation was less than 7%. EPR spectra were recorded on an EMXnano EPR spectrometer at room temperature and in the dark. Blue line: fresh sample, red line: sample that has been stored on ice for 3 h in amber tubes

To determine the stability of WB in MilliQ water, we measured the mass spectra of a fresh solution (150 min after preparation) and a solution that had been prepared and then stored at room temperature for 21 days in amber tubes. Whereas the mass spectrum of the sample stored in aqueous solution for 150 min clearly showed the presence of WB (*m/z* = 164.131), the spectrum of the dissolved sample stored for 21 days showed only traces of WB (6%), but mostly a signal at 144.984 *m/z* in addition to a signal at 112.958 *m/z* (Table S9 and Figure S7) that could not be identified. We obtained similar results for different storage conditions using UV–Vis spectroscopy; here, the decay of the radical cation can be monitored by its decoloration [12]. The blue-colored radical cation exhibits absorbance maxima around 560 nm and 610 nm and the extinction coefficient of the latter wavelength was used to calculate the specific enzymatic activity of MDH in kinetic assays [12, 57]. EPR and UV–Vis measurements (Fig. 6) confirmed a good stability of the WB radical in MilliQ water (A) and in buffered solution of pH 7 (data not shown) as well as in samples that had been briefly stored on ice in MilliQ water and were diluted in alkaline buffer just before analysis (6B). An alkaline pH led to a fast degradation of the WB radical (Fig. 6c). Since the radical cation has been reported stable in aqueous solutions at a pH of 3.5–6 but undergoes degradation outside this pH range [55] and under routinely used assay conditions (pH 9), a prolonged incubation of the dye under conditions of high pH should be avoided.

2. Storage temperature

**Fig. 7:**
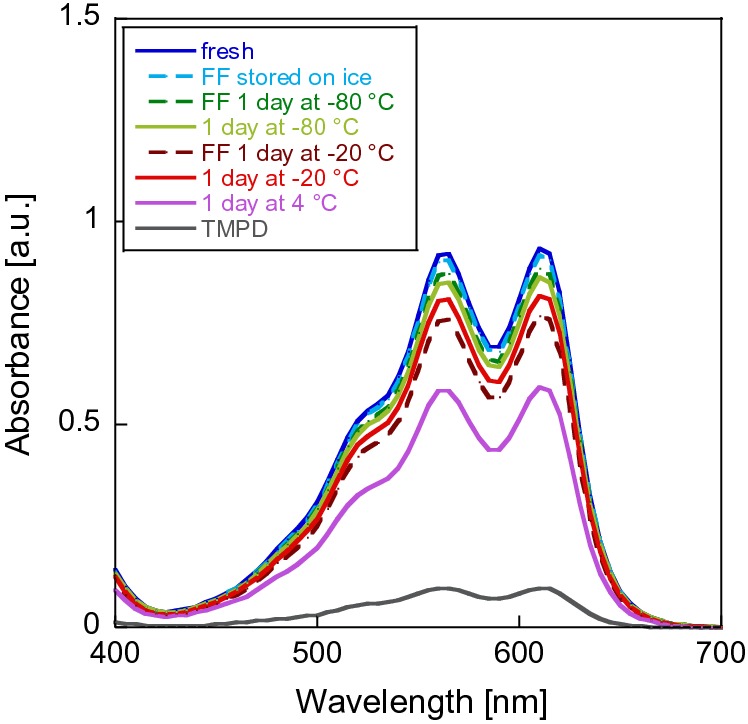
UV–Vis spectra of differently stored WB in 100 mM multicomponent buffer pH 9. 2 mM WB samples were stored in MilliQ water and diluted with buffer to a concentration of 200 µM before measurement. Spectra of triplicates were recorded at 30 °C on an Epoch2 plate reader without path length correction. The buffer baseline was subtracted from the spectrum. The standard deviation was less than 10%. (FF, flash frozen)

Next, we analyzed WB samples that were stored under different conditions in MilliQ water and were either flash frozen in liquid nitrogen or frozen slowly before storage on ice (Fig. 7). Flash freezing did not influence the radical cation concentration, whereas the storage temperature had a major effect. We found the best storage temperature to be − 80 °C, and higher temperatures of − 20 °C resulted in a decrease of the radical cation. Storage at 4 °C for 24 h nearly halved its concentration. Additionally, storage at room temperature for 3 weeks (see Supporting Information Table S9 and Figure S7 for more details) led to almost complete decomposition as shown in MS-experiments. We, thus, suggest avoiding the storage of WB solutions.

3. Effect of pH and temperature

**Fig. 8:**
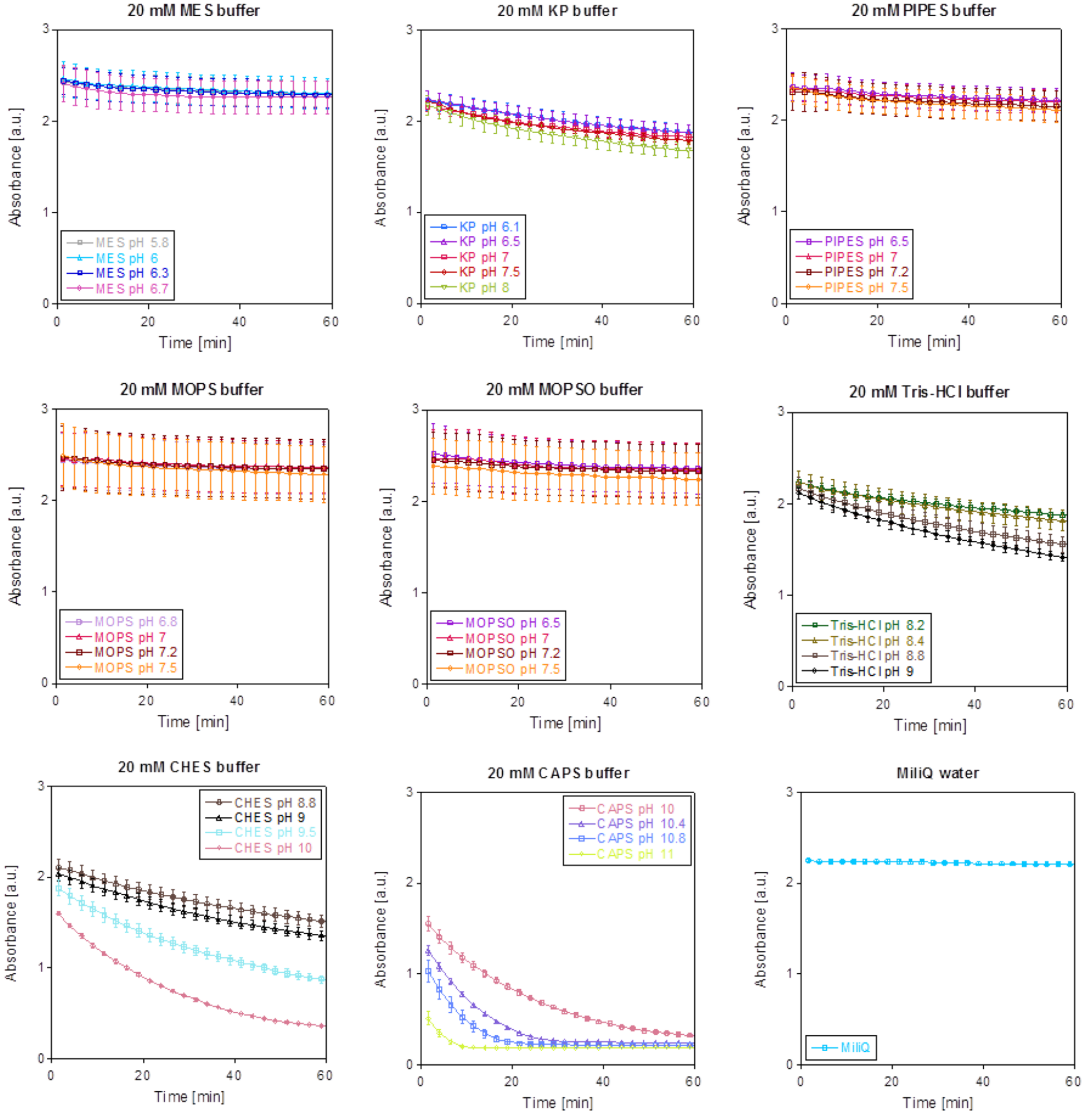
pH dependence of WB in different buffers. Conditions were as follows: 200 µM WB in 20 mM buffer of different pH, heated for 1 h at 30 °C. Absorbance at 610 nm was monitored with an Epoch2 plate reader. Experimental and technical (CHES and CAPS) triplicates with standard deviations are shown. Data were path length corrected to 1 cm

We further evaluated the effect of the assay condition (pH, buffer system and temperature) on WB radical cation stability. Our results (Fig. 8) reveal that organic buffers with acidic and neutral pH such as MES, MOPS and MOPSO do not influence the radical cation stability negatively. Yet, PIPES buffer caused a slight decrease in absorbance over time, which was more pronounced in the inorganic potassium phosphate buffer. Further, buffers of alkaline pH such as CAPS or CHES led to a fast decomposition of the dye. Moreover, compared to a temperature of 45 °C (data not shown), the WB absorbance was more stable at 30 °C.

The negative effects of high temperature and pH on WB decomposition are also corroborated by EPR spectroscopy (Figure S8). We therefore performed the following kinetic assays at 30 °C. Interestingly, when aqueous solutions of the precursor TMPD or the dichloride salt TMPDD were heated to 45 °C, formation of the Wurster’s blue radical was observed to some extent, depending on the pH of the buffer system (Figures S9 and S10).

4. WB in MDH assays

**Table 2:** Extinction coefficients of WB in different buffer systems, at different pH values and temperature

Wavelength (nm)	Extinction coefficient ε_610nm_ (mM^−1^ cm^−1^)	Buffer system	pH	Temperature (°C)
560	12.30 [48]	100 mM CHES	9	30
600	9.00 [71]	64 mM sodium borate	9	22
600	9.00 [11]	100 mM tetrasodium pyrophosphate	9	22.5
610	9.75 ± 0.48	Multicomponent buffer^a^	6.2	30
610	9.60 ± 0.34	Multicomponent buffer^a^	7.0	30
610	9.53 ± 0.34	Multicomponent buffer^a^	7.5	30
610	9.51 ± 0.26	Multicomponent buffer^a^	8.1	30
610	8.82 ± 0.37	Multicomponent buffer^a^	9.0	30
610	9.67 ± 0.41	Multicomponent buffer^b^	7.0	30
610	8.17 ± 0.41	Multicomponent buffer^b^	9.0	30
612	12.70 [12]	50 mM MOPSO/50 mM CHES	7/9	20
640	2.14 [17]	100 mM Sodium tetraborate	9	–
640	2.78 [48]	100 mM CHES	9	30
652	1.07 [12]	50 mM MOPSO/50 mM CHES	7/9	20

^a^Multicomponent buffer: 2.5 mM citric acid, 2.5 mM Bis–Tris, 2.5 mM Tris and 2.5 mM CHES

^b^Multicomponent buffer: 25 mM citric acid, 25 mM Bis–Tris, 25 mM Tris and 25 mM CHES

**Fig. 9:**
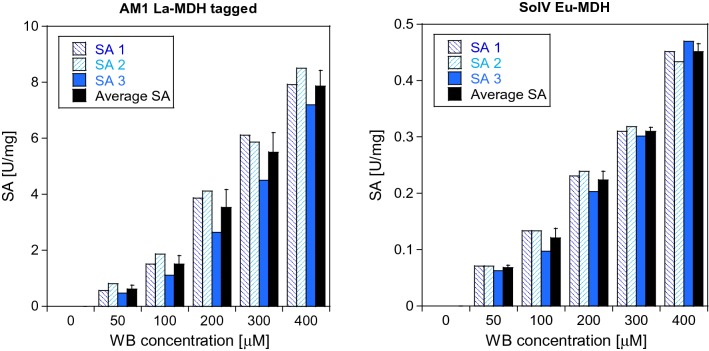
WB dependence of AM1 La-MDH and SolV Eu-MDH. The specific activity (SA, in μmol min^−1^ mg^−1^) of His-tagged AM1 La-MDH (left) was determined in 100 mM multicomponent buffer, pH 9, with 15 mM NH_4_Cl. SolV Eu-MDH activity (right) was measured in 100 mM multicomponent buffer, pH 7.2, with added 20 µM Eu(III). The WB concentration was varied, and protein concentration was constant at 100 nM for AM1 La-MDH and 200 nM for SolV Eu-MDH. The assay was performed with 50 mM MeOH at 30 °C and 610 nm. The total volume in wells was 200 µL. All SA are technical replicates. SA1 and SA2 were determined by different pairs of hands than SA3. Data were collected at an Epoch2 plate reader

Our insights regarding the stability and handling of WB were verified using La-MDH from *M. extorquens* AM1 (Figure S11). We confirmed that flash freezing the dye in liquid nitrogen preserved the WB solution and thus did not affect MDH specific activity (SA) negatively, whereas storage of WB stocks at 4 °C led to a decreased SA even after adjustment of the WB concentration. In contrast, the MDH activity was restored by concentration adjustment in WB samples that have been stored in MilliQ water at − 20 °C and − 80 °C and shows only slight variations within the error range. Additionally, the precursor of WB, TMPD, was tested as EA for MDH. But both TMPD and a mixture of WB and TMPD led to no or decreased methanol oxidation by MDH. Next, the WB concentration dependence of both AM1 La-MDH and SolV Eu-MDH was analyzed (Fig. 9). Both MDH types showed increasing SA in the range of 0–400 µM WB and a linear WB dependence. SolV Eu-MDH exhibited a notably lower enzymatic activity, which is likely due to the impact of Eu^3+^ on catalytic efficiency [19]. Also, a temperature of 30 °C instead of 45 °C was used, which was less than optimal for this MDH. In the case of SolV Eu-MDH, no WB inhibition occured at 400 µM, so higher concentrations can be used [9]. For a better comparability of the two MDH types, we chose a WB concentration of 200 µM.

Further, the influence of ammonia/ammonium ions on the activity of AM1 La-MDH (Figure S12) with WB was studied [9, 16]. Both the free ammonia base and the ammonium ion were reported to either positively or negatively influence (as activator or inhibitor) MDH, but this mechanism is still not fully understood [8, 58, 59]. SolV Eu-MDH shows activity without an additional activator (data not shown) [4, 19, 60]. Activity was low for both polyhistidine tagged and untagged AM1 La-MDH in the absence of NH_4_Cl, while the addition of 15 mM NH_4_Cl led to the highest enzymatic activity in the range studied (Figure S12). Taken together, our results show that WB can be utilized as a single reagent EA/dye for Ln-MDH assays. The step-by-step assay procedure and handling suggestions for the use of WB as electron acceptor are described in the Supporting Information as well as summarized in Table 3 below. To sum up, prolonged storage, high temperatures and pH should be avoided, if possible, as these parameters lead to rapid degradation of WB. If additives such as metal ions or ammonia are required, WB stability under the new conditions should be evaluated first without added enzyme. Using a plate reader, a concentration of 200 µM WB for routine assays presents a good starting point.

**Table 3:** Handling suggestions of artificial electron acceptors for MDH assays

Stage	Step no	Description	Note
Handling suggestions for PES/PMS DCPIP assay			
Stock solution preparation	1	Prepare a 100 mM PMS/PES stock solution in MilliQ water	Exclude light, stock solution should be made fresh in amber-colored tubes and stored on ice until measurement
	2	Prepare a 2 mM DCPIP stock solution in MilliQ water	Exclude light, stock solution should be made fresh in amber-colored tubes and stored on ice until measurement
	3	Prepare additives for the assay (e.g., EuCl_3_ or NH_4_Cl stock solutions)	
Determine extinction coefficient (ε_600_) for DCPIP under chosen conditions	4	Use same buffer system (type of buffer and concentration, pH, temperature as for the assays)	Temperature can affect the pH of certain buffers significantly
Assay mix preparation	5	Mix PMS/PES and DCPIP stock solutions in buffer to a final concentration of 100 µM DCPIP and 1 mM PMS/PES	Assay mix should be heated for 15 min at 45 °C in the dark
Spectrophotometric read-out	6	Mix assay mix with MDH/MeOH in a 96-well plate and equilibrate 2 min at assay temperature	Minimize light exposure and monitor the background of the assay mix at 600 nm
	7	Add MeOH/MDH to start the assay	Minimize light exposure
Handling suggestions for WB assay			
Stock solution preparation	1	Prepare a 1 mM WB stock solution in MilliQ water	Low solubility limit, exclude light, stock solution should be made fresh in amber-colored tubes and used immediately
Determine extinction coefficient (ε_610_) for WB under chosen conditions	2	Use same buffer system (type of buffer and concentration, pH, temperature as for the assays)	Temperature can affect the pH of certain buffers significantly
Assay mix preparation	3	Mix WB with buffer to a final concentration of 200 µM	Alkaline pH leads to a fast decomposition of WB, exclude light
Spectrophotometric read-out	4	Mix WB/buffer with MDH/MeOH in a 96 well plate and equilibrate 2 min at assay temperature	Lower temperatures are preferable, minimize light exposure and monitor background at 610 nm
	5	Add MeOH/MDH to start the assay	Minimize light exposure. Monitor decomposition of the dye

## Conclusions

In this work, we present a thorough analysis of the buffers, electron acceptors PMS and PES and the redox dyes DCPIP and WB used in MDH assays. We provide recommendations for the handling of these compounds to minimize decomposition and unwanted side reactions in the absence of MDH. Most importantly, radical formation of the EA, leading to a non-enzymatic reduction of DCPIP in MDH assays, can be minimized through the exclusion of light. Overall, PMS is more prone to degradation than PES. Further, the one-electron acceptor and redox dye, WB, was used for the first time in assays with lanthanide-dependent MDH. This radical cation was synthesized from TMPDD using bromine and found to be best suited for a quick identification of enzymatic activity at a concentration of 200 µM. A summary of the most important handling suggestions is provided in Table 3.

In summary, the PES (or PMS) and DCPIP coupled assay is the method of choice for MDH kinetic analysis and can yield reproducible results when the components are handled correctly. Parameters determined with this artificial assay (originally developed by Anthony and Zatman) such as pK_a_ values, pH dependence or Arrhenius activation energies from temperature-dependence measurements are similar to the ones determined from protein electrochemistry when using the natural electron acceptor cytochrome *c*_GJ_ [14]. The one-electron acceptor and dye WB, on the other hand, presents an easy method for routine MDH assays, for example, identifying MDH containing fractions during enzyme purification. Due to its low stability at alkaline pH, PES-DCPIP is preferable to WB as EA/dye for determining the kinetic parameters. With this study we aimed to provide information about the handling of electron acceptors used in MDH assays to promote consensus in assay measurements for better comparability of results.

## Electronic supplementary material

Below is the link to the electronic supplementary material.

Supplementary file1 (PDF 1047 kb)
