# Supplementary material for: Understanding the chemistry of the artificial electron acceptors PES, PMS, DCPIP and Wurster’s Blue in methanol dehydrogenase assays
Source: J Biol Inorg Chem. 2020 Feb 14;25(2):199–212. doi: 10.1007/s00775-020-01752-9 (PMC7082304; doi:10.1007/s00775-020-01752-9)
Supplement: Supplementary file 1 — Supplementary file1 (PDF 1047 kb) [file 775_2020_1752_MOESM1_ESM.pdf]

Supporting Information

Understanding the chemistry of the artificial electron acceptors PES, PMS, DCPIP and Wurster's Blue in methanol dehydrogenase assays

Berenice Jahn,[a] *Niko S.W. Jonasson,*[a] *Hurina Hu,*[a] *Helena Singer,* [a] *Arjan Pol,*[b] *Nathan M. Good*[c]*, Huub J. M. Op den Camp*[b]*, N. Cecilia Martinez-Gomez*[c] *and Lena J. Daumann*[a,*]

[a]Department of Chemistry, Ludwig-Maximilians-Universität München, Butenandtstr. 5-13, 81377 München, Germany.

[b]Department of Microbiology, Institute of Wetland and Water Research, Radboud University, Nijmegen, The Netherlands.

[c]Department of Microbiology and Molecular Genetics, Michigan State University, East Lansing Michigan, USA.

Materials and Methods

Materials

Table S1 Electron acceptors

Compound	Abbreviation	Purity	Supplier	Note
2,6-dichlorophenolindophenol sodium salt hydrate	DCPIP	≥ 90%	Sigma-Aldrich	
N,N,N´,N´-tetramethyl-p-phenylenediamine	TMPD	99%	Sigma-Aldrich	
N,N,N´,N´-tetramethyl-p-phenylenediamine dihydrochloride	TMPDD	≥ 97%	Sigma-Aldrich	
phenazine methosulfate	PMS1	≥ 90%	Sigma-Aldrich	Lot# 125M4099V, yellow
	PMS2	≥ 90%	Sigma-Aldrich	Lot# 057M4019V, yellow
	PMS3	98%	abcr	Lot# 1385955, grey yellow
phenazine ethosulfate	PES1	≥ 95%	Sigma-Aldrich	Lot# MKCD9943, brown, opened for eleven months
	PES2	≥ 95%	Sigma-Aldrich	Lot# MKCD9943, brown, opened for seven months
	PES3	≥ 95%	Sigma-Aldrich	Lot# MKCD9943, brown, opened for five months
	PES4	≥ 95%	Sigma-Aldrich	Lot# MKCG3495, orange, opened for six months

Table S2 Buffers

Buffer	Abbreviation	Purity	Supplier
bis(2-hydroxyethyl)amino-tris(hydroxymethyl)methane	BIS-TRIS	BioXtra, ≥ 98%	Sigma-Aldrich
3-(Cyclohexylamino)-1-propanesulfonic acid	CAPS	BioXtra, ≥ 99%	Sigma-Aldrich
2-(Cyclohexylamino)ethanesulfonic acid	CHES	≥ 99%	Sigma-Aldrich
2-(N-Morpholino)ethanesulfonic acid hydrate	MES	≥ 99.5%	Sigma-Aldrich
3-(N-Morpholino)propanesulfonic acid	MOPS	BioPerformance certified, ≥ 99.5%	Sigma-Aldrich
3-Morpholino-2-hydroxypropanesulfonic acid	MOPSO	≥ 99%	Sigma-Aldrich
1,4-Piperazinediethanesulfonic acid	PIPES	BioPerformance certified, ≥ 99%	Sigma-Aldrich
potassium phosphate tribasic	KP	reagent grad, ≥ 98%	Sigma-Aldrich
tris(hydroxymethyl)aminomethane	Tris	ultrapure grade, ≥ 99.9%	Sigma-Aldrich

Table S3 Reagents for the synthesis of WB and pyocyanin

Reagent	Purity	Supplier
Bromine	puriss. p. a. ≥ 99%	Sigma-Aldrich
Chloroform	HPLC grade	Fisher Chemicals
Diethylether	99.5% ACS	Bernd Kraft
Isohexane	-	Fisher Chemicals
sodium carbonate	analytical reagent	VWR chemicals
sodium perchlorate monohydrate	reagent grad	Fisher Chemicals

Table S4 Assay components

Component	Purity	Supplier
ammonium chloride	-	VWR chemicals
europium(III) chloride hexahydrate	99.99%	Sigma-Aldrich
Methanol	HPLC grade	Fisher Chemicals
potassium cyanide	96.00%	VWR chemicals

Methods

Synthesis of N,N,N',N'-tetramethyl-p-phenylenediamine perchlorate (Wurster's Blue, WB, 4b) according to a modified literature procedure.[1] As a precaution and because the WB synthesis protocol involves bromine, an aqueous sodium thiosulfate solution (158.11 g, in 1 L H_2O) was prepared first for quenching residual or spilled solutions. It is also important that all components are fully dissolved before they are added together. In a round bottom flask equipped with a pressure equalizing dropping funnel N,N,N′,N′-tetramethyl-p-phenylenediamine dihydrochloride (**4a**, 1.00 g, 4.22 mmol, 1.00 equiv.) was dissolved in 18 mL water (MilliQ–grade) at room temperature. A solution of sodium perchlorate (3.65 M in methanol, 28.0 ml, 12.50 g, 102.09 mmol, 24.00 equiv.) was added to the clear pale pink-colored solution in the round bottom flask. Meanwhile, an aqueous bromine solution was prepared by dissolving 100 µl Br_2 in 32 mL of MilliQ water by stirring for about 15 min and added to the dropping funnel. The flask was then cooled to -10 °C using isopropanol and dry ice. While maintaining a bath-temperature between -9 °C and -13 °C the bromine solution (0.063 M, 2.01 mmol, 0.50 equiv.) was added dropwise (1 drop per second) to the reaction mixture in the round bottom flask, gradually resulting in a deep blue solution and a blue precipitate. After warming to 10 °C, the precipitate was filtered off using a sintered glass frit (porosity 4) and washed with ice-cold methanol (2 x 10 mL) and then diethyl ether (2 × 2 mL). The deep blue-brown powder was dried *in vacuo* to yield WB as a brown-metallic powder (Yield 0.54 g, 2.06 mmol, 49%). Storage at room temperature under nitrogen showed no sign of degradation over weeks. Storage at room temperature for several months, however, is not recommended. Elemental microanalysis showed small impurities of sodium perchlorate, and recrystallization via diethyl ether vapor diffusion into a methanolic solution of WB can be carried out, this step however introduces additional traces of methanol (MDH substrate). **FT-IR spectroscopy**: $\tilde{\nu}$ = 1540, 1419, 1379, 1341, 1229, 1069, 975, 942, 825 cm^{-1}. **HR-MS (ESI +)** Calculated for $[C_{10}N_2H_{16}]^{\cdot+}$: 164.1314 m/z. Found: 164.1308 m/z. **Elemental Analysis:** Calculated for $[C_{10}N_2H_{16}]^{\cdot+}$ $[ClO_4]^{-}$: C 45.55, H 6.12, N 10.62. Found: C 45.12, H 5.93, N 10.34.

Synthesis of pyocyanin via the photochemical oxidation of phenazine methosulfate was conducted according to a modified literature procedure.[2] Phenazine methosulfate (50 mg, 0.16 mmol, 1.00 equiv.) was dissolved in 50 mL water (MilliQ–grade). The yellow solution was exposed to daylight for 36 h resulting in a deep red solution. Sodium carbonate solution (1.58 M in water, 0.500 ml, 0.79 mmol, 5.00 equiv.) was added at once, turning the solution turquoise. The solution was exposed to daylight for another 6 h and 1 mL of an aqueous sodium carbonate solution (10%) was added. The turquoise solution was extracted with chloroform (3 × 100 ml), dried over saturated sodium chloride solution and potassium carbonate. The solvent was reduced to a total volume of 20 mL using a rotary evaporator (250 mbar, 40 °C). Crystallization from distilled *iso*-hexane via vapor diffusion was carried out to yield the product as deep blue needles (20 mg, 0.095 mmol, 60%). **^{1}H NMR (400 MHz, CD_3OD, 295 K):** δ = 8.30 (s, 1H), 8.09-7.95 (m, 2H), 7.94-7.83 (m, 1H), 7.69 (t, *J* = 7.6 H, 1H), 6.54 (dd, *J* = 33.0 Hz, *J* = 8.6 Hz, 2H), 4.19 (s, 3H) ppm. **^{13}C NMR (100 MHz, CD_3OD, 295 K):** δ = 144.9, 135.7, 132.0, 125.5, 114.8, 113.9, 92.8, 34.4 ppm. These chemical shifts were collected from HMQC measurements as the signals in a normal ^{13}C NMR spectrum could not be distinguished from the background noise. **HR-MS (ESI+):** Calculated for $C_{13}H_{11}N_2O$ $[M+H]^+$: 211.087 m/z. Found $[M+H]^+$: 211.086 m/z

Investigation of the electron acceptors

Uv-Vis spectra of DCPIP and Wurster´s Blue: 2 mM stock solutions of both dyes were prepared in MilliQ water. WB was shaken for 1 h at room temperature and afterwards put into an ultrasonic bath for 5 min. The stock solutions were diluted in 100 mM multicomponent buffer pH 7.2 and 9 respectively to a concentration of 50 µM (DCPIP) or 100 µM (WB). Spectra were recorded at a Cary60 spectrophotometer at room temperature while stirring.

Analysis of N,N,N´,N´-tetramethyl-p-phenylenediamine derivatives: For the time-dependent experiment, stock solutions of 2.5 mM N,N,N´,N´-tetramethyl-p-phenylenediamine (TMPD), 2.5 mM N,N,N´,N´-tetramethyl-p-phenylenediamine dihydrochloride (TMPDD, **4a**) and 2 mM of the synthesized WB radical cation (**4b**) were prepared in MilliQ water in amber tubes. For spectrophotometric analysis 200 µM samples were prepared by diluting the stock solutions with different buffers (MES, potassium phosphate (KP), PIPES, MOPSO, MOPS, Tris-HCl, CHES, CAPS or MilliQ water) of different pH and a concentration of 20 mM directly in the 96 well plate. All measurements were carried out at an Epoch2 plate reader at 610 nm and either 30 °C or 45 °C for 1 hour. Data were automatically path length corrected to

1 cm with the Gen5 software. For storage experiments, a 2 mM WB stock solution in MilliQ water was prepared. The samples were either analyzed just after preparation or were stored on ice, flash frozen in liquid N_2 for about 20 seconds and let thaw on ice immediately in the case of the “FF on ice sample” or stored at -20 °C or -80 °C or were normally frozen at -20 °C or -80 °C. Samples that were stored on ice were either diluted with MilliQ water or 100 mM multicomponent buffer pH 9. Samples were thawed on ice before analysis if frozen and diluted to a concentration of 200 µM in either MilliQ water, 100 mM multicomponent buffer pH 7.2 or pH 9, 20 mM potassium phosphate pH 7.2 or 20 mM Tris-HCl pH 9. Measurements were carried out with an Epoch2 plate reader. Spectra were collected at 30 °C and a separately recorded MilliQ water or buffer baseline was subtracted.

Mass Spectrometry: Mass spectra were collected on a Thermo Fisher Orbitrap Q Exactive®. A 50:50 mixture of water and methanol was used as eluent.

PMS was dissolved in unbuffered water and exposed to eight different conditions in two sets of experiments of four samples each: aerobic (O_2 was added) and anaerobic (samples strictly handled under an atmosphere of nitrogen, degassed water was used). Two samples of each set were kept in the dark whereas the other two samples were exposed to daylight. Of each of the two sample sets, one was cooled to 0 °C whereas the other was heated to 45 °C. Samples were taken after 1 h and 2.5 h, diluted and stored at 0 °C in the dark until they were injected into a Thermo Fischer Orbitrap. In these measurements up to four distinct signals were detected: 188.128 m/z, 195.092 m/z, 210.110 m/z, and 211.086 m/z (Figure 2). A complete report of the signals is shown in Table S5. The predominant signal was given 100% abundance and all other signals were normalized relative to this. No direct quantitative evaluation can be performed due to possibly different ionization potentials; however qualitative trends can be observed and described. In a second series of mass spectrometry experiments we investigated the influence of the individual components of the DCPIP/PMS-coupled assay. MDH enzymes are often supplemented with potassium cyanide for stability. Moreover, ammonia is often added to the assay as an activator.[3] However, both cyanide and ammonia have been reported to react with PMS.[2, 4] Here, we incubated PMS with ammonia and cyanide at room temperature, both in the dark and in daylight. Samples were collected after 100 min and 300 min and analyzed using mass spectrometry. Across all samples, four distinct signals were detected: 181.076 m/z, 195.092 m/z, 210.089 m/z, and 211.086 m/z (Table S6). In addition to the four signals mentioned above, in the case of the reaction of PMS with potassium cyanide at room temperature in daylight, a signal was detected at 220.087 m/z which can be ascribed

to the structure shown in Figure 2 (**1e**). This signal was observed as a minor peak after 100 min and 300 min with around 40-50% relative abundance. A signal corresponding to 1d in Figure 2, as has been described in the literature,[2] was detected in the reaction of PMS with ammonia in light after 100 min and 300 min, though only as a minor signal with a relative abundance of 6% and 16%, respectively. When light was excluded neither of these two signals was detected, again highlighting the importance of preventing light exposure to the assay mixture during handling. In all cases the signal at 181.076 m/z was detected as a major peak, corresponding to the demethylation product phenazine (**1b**), as was shown with the reference sample mentioned above. Additionally, a signal at 211.086 m/z was detected in all samples, although this signal was most prevalent in the light-exposed experiments. This signal most likely corresponds to protonated pyocyanin (**1c**), which would be in accordance with the literature[2] and the data presented above.

We also investigated the decomposition of PES under the influence of daylight. Similar to our experiments with PMS, we exposed aqueous solutions of PES to daylight and performed control reactions in the dark, both at room temperature and at 0 °C. As can be seen in Table S7, our data fits the previously published observations: PES decomposes mainly to a species with 225.102 m/z ($[M+O]^+$) while phenazine is detected only in traces. The observed signal at 225.102 m/z most likely corresponds to an ethyl-pyocyanin derivative (**2b**).

Measurement of a complete assay mix sample (1 mM PES, 100 µM DCPIP, and 20 µM $EuCl_3$ in 20 mM PIPES) The sample was conditioned at 45 °C for 15 min and always kept in the dark, as it would have been if it were to be used for assay experiments with MDH enzymes. As can be seen in Figure S1, hardly any oxidation (~1%) and no de-ethylation of PES can be observed under these conditions.

EPR spectroscopy: The radical formation in aqueous PES, PMS and WB samples was analyzed using EPR spectroscopy. PMS/PES: Stock solutions of 100 mM PES or PMS in MilliQ water were diluted in MilliQ water or 100 mM multicomponent buffer of pH 7.2 or 9 or 20 mM PIPES buffer pH 6.2 or pH 7.2 or 20 mM KP buffer pH 7.2 to a concentration of 10 mM. Samples were treated differently (storage in the dark at room temperature or 4 °C in the fridge for about 30 min, heating at 45 °C for 15 min in amber tubes, exposure to daylight or UV light of 254 nm for 5 min each). WB: A 2 mM WB stock solution in MilliQ water was diluted in 100 mM multicomponent buffer of pH 7.2 or 9 to a concentration of 200 µM. Both PMS/PES and WB samples were analyzed subsequently after preparation. Samples were

picked up using a capillary (BLAUBRAND intraMARK 50 µl), that was sealed with wax afterwards. EPR spectra were recorded at room temperature at an EMXnano EPR spectrometer in the dark. The PMS and PES samples were prepared and analyzed on both a sunny and a cloudy day whereas WB samples were analyzed on a sunny day only.

MDH assays

Eu-MDH from *Methylacidiphilum fumariolicum* SolV (SolV-MDH, UniProt ID: I0JWN7) and La-MDH from *Methylorubrum extorquens* AM1 (AM1-MDH, UniProt ID: C5B120) were purified as described previously.[5] AM1-MDH was both tested with and without polyhistidine-tag and this is indicated by U (untagged) or T (tagged), respectively.

A **washing procedure** was used for removal of degraded protein and storage methanol, formaldehyde and/or formic acid from the enzyme and for re-buffering purposes. Each protein sample was diluted in chelexed buffer in a 1:10 ratio before centrifugation. The diluted protein was centrifuged at 4,500 rpm and 4 °C until the initial volume was reached using a filter unit with a 30 kDa molecular weight cut-off. The procedure was repeated twice.

Determination of the protein concentration via the Edelhoch method: The protein concentration was measured with the photometric microvolume analysis using a TrayCell (Hellma Analytics, Germany) equipped with a 10x cap in a Cary60 (Agilent Technologies, CA, USA) UV-vis spectrometer. A buffer baseline was recorded separately. The absorption at 280 nm of the protein and a previously determined MDH specific factor were utilized for calculating the protein concentration. The following specific factors were determined and used: 1.46 for the AM1 La-MDH and 2.56 for the SolV Eu-MDH.

Spectrophotometric determination of SolV MDH activity with PMS/PES: All of the used stock solutions for this experiment were prepared in MilliQ water and were further diluted in buffer before the experiment. Two types of experiments were performed. For the analysis of the specific activity of MDH at 30 °C 100 mM multicomponent buffer pH 7.2 (consisting of 25 mM citric acid, 25 mM Bis-Tris, 25 mM Tris-HCl and 25 mM CHES) was utilized whereas for the experiment at 45 °C chelexed 20 mM PIPES buffer pH 7.2 was used. A 1 mM solution of DCPIP was mixed with either 10 mM PMS or 10 mM PES. When the previously used assay conditions were applied, the mixture of DCPIP and PMS or DCPIP and PES was additionally incubated in a dry block heater for 15 min at 45 °C and 1 mM KCN was included in the assay, which was performed at 45 °C. Afterwards, the electron acceptors, 1 M methanol

(10 µl) and the corresponding buffer were added into a 96 well plate. The plate was incubated for 2 min at 30 °C or 45 °C in an Epoch2 plate reader. Washed SolV-MDH was mixed with buffer and 2 mM $EuCl_3$ (2 µl) and was added into the plate. The decrease in absorbance was followed at 600 nm for 20 min. The total volume of 200 µl/well consisted of 100 nM Eu-MDH, 20 µM $EuCl_3$, 50 mM MeOH, 100 µM DCPIP and 1 mM PMS or PES. For the background control the absorbance at 600 nm was monitored as described above except that no MDH was added. The obtained values were automatically path length corrected to 1 cm using the Gen5 software. For calculating initial rates, the first 5 min of the slope were taken.

Spectrophotometric determination of AM1-MDH activity with PMS/PES: All of the stock solutions used for this experiment were prepared in MilliQ water and were further diluted in buffer before the experiment. A 1 mM solution of DCPIP (20 µl) was mixed with either 10 mM PMS or 10 mM PES (20 µL), 1 M methanol (10 µL) and 100 mM multicomponent buffer pH 9 (consisting of 25 mM citric acid, 25 mM Bis-Tris, 25 mM Tris-HCl and 25 mM CHES) in a 96 well plate. The plate was incubated for 2 min at 30 °C in an Epoch2 plate reader. Washed AM1 La-MDH was mixed with 500 mM NH_4Cl (6 µL) and buffer and was added to the assay mix in the plate. The decrease in absorbance at 30 °C was followed at 600 nm for 15 min. The total volume of 200 µL/well consisted of 100 nM La-MDH, 15 mM NH_4Cl, 50 mM MeOH, 100 µM DCPIP and 1 mM PMS or PES in 100 mM multicomponent buffer pH 9. For the background control a 96 well plate was prepared as described above except that no MDH was added. The obtained values were path length corrected. For calculating the initial rate, the first 1 min of the slope was used.

Spectrophotometric determination of MDH activity with WB: All of the used stock solutions were prepared in MilliQ water and were further diluted in buffer before the experiment. A 2 mM WB solution was prepared by dissolving the synthesized WB perchlorate salt for 20 min to 1 h at room temperature in an aluminum foil-wrapped tube on a shaker. The time to dissolve WB varied between experiments and was dependent on the particle size of the dye. Further solutions were 500 mM NH_4Cl (for AM1 La-MDH) or 2 mM $EuCl_3$ (for SolV Eu-MDH) and 1 M MeOH. Two different buffers were used: 20 mM Tris-HCl pH 9 and 100 mM multicomponent buffer pH 7.2 and pH 9. 100 µL (half of the assay volume) containing WB (the concentration of WB was kept constant or was varied depending on the experiment type) and the corresponding buffer were added into the well. Afterwards 1 M methanol (10 µL) was added. The mixture was incubated for 2 min at 30 °C in the plate reader. Meanwhile MDH was mixed with either NH_4Cl (AM1 La-MDH) or $EuCl_3$ (SolV Eu-

MDH) and buffer and was then added to the assay mix. In the case of AM1 La-MDH the final assay mix contained 100 nM MDH, 15 mM NH_4Cl, 200 µM WB and 50 mM methanol. For the assay with SolV Eu-MDH 200 nM MDH, 20 µM Eu, 200 µM WB and 50 mM methanol were used per well. Path length-corrected data were collected at 30 °C and 610 nm using an Epoch2 plate reader. For calculating the initial rate, the first 30 s (AM1 La-MDH) or first 5 min (SolV Eu-MDH) of the slope were used together with the determined extinction coefficient for the given assay condition (pH, temperature, buffer system, Table 2). Since WB is a one-electron acceptor and two molecules of WB are needed per molecule of MeOH, the SA was divided by two. Normalized WB SA were similar to SA determined with PES/PMS.

FT-IR spectroscopy of PMS and PES samples: Solid PMS and PES samples were analyzed at room temperature using a Jasco FT/IR-460 Plus with a ATR Diamond element. The following batches were examined. PMS samples (Supplier, Lot#, appearance): (PMS1) Sigma Aldrich, 125M4099V, yellow; (PMS2) Sigma Aldrich, 057M4019V (yellow), (PMS3) abcr Lot# 1385955 (grey yellow). PES samples (Supplier, Lot#, appearance): (PES1) Sigma Aldrich Lot# MKCD9943 (brown) opened for eleven months, (PES2) Sigma Aldrich Lot# MKCD9943 (brown) opened for seven months, (PES3) Sigma Aldrich Lot# MKCD9943 (brown) opened for five months, (PES4) Sigma Aldrich Lot# MKCG3495 (orange) opened for six months. It can be observed, that the abcr® sample (>98% purity, PMS3) is missing a peak at 1435 cm^{-1}, shows additional and red-shifted peaks in the region of 1030-780 cm^{-1} and a different signature between 1300-1100 cm^{-1} and 750-650 cm^{-1} compared to the IR-spectra of Sigma Aldrich® samples PMS1 and PMS2 (>90% purity, Figure S2).

UV-vis spectroscopy of PMS and PES samples: 10 mM stock solutions in MilliQ water were prepared and diluted to a concentration of 125 µM in MilliQ water. Spectra were recorded after sample preparation at room temperature using an Epoch2 plate reader. Afterwards samples were exposed to daylight for 1 day and spectra were recorded again under the same conditions. Batches were as described above. The UV-Vis spectrum of the light-exposed abcr® sample (PMS3, Figure S3) exhibits an entirely different spectrum and a blue shift of the peak at 700 nm to 520 nm compared to PMS1 and PMS2.

UV-vis spectra of MDH samples: Both SolV Eu-MDH and untagged AM1 La-MDH were washed twice in 20 mM PIPES buffer pH 7.2 and concentrated to a concentration of 15 µM for Eu-MDH and 7 µM for La-MDH. Spectra were recorded at a Cary60 spectrophotometer at room temperature.

Supporting Tables

Table S5 Results of the exposure of PMS to the conditions described in Figure 2.

reaction time [h]	Temperature [°C]	m/z signals	Relative Abundance [%]			
			aerobic		anaerobic	
			light	dark	light	dark
1	0	188.128	7	3	23	19
		195.092	96	100	100	100
		210.110	7	0	40	33
		211.086	100	5	4	6
	45	188.128	78	12	70	40
		195.092	30	100	79	100
		210.110	46	12	100	70
		211.086	100	4	63	78
2.5	0	188.128	7	3	14	15
		195.092	3	100	52	100
		210.110	5	1	23	31
		211.086	100	7	100	8
	45	188.128	28	2	23	17
		195.092	6	100	11	100
		210.110	23	0	28	27
		211.086	100	5	100	7

Table S6 Results of the reaction of PMS with cyanide and ammonia.

reaction time [min]	Reactant	m/z signals	Relative Abundance [%]	
			light	dark
100	KCN	181.076	30	98
		195.092	15	100
		210.089	13	23
		211.086	100	18
	NH_3	181.076	71	100
		195.092	4	34
		210.089	0	0
		211.086	100	9
300	KCN	181.076	95	100
		195.092	23	0
		210.089	31	1
		211.086	100	7
	NH_3	181.076	91	100
		195.092	0	0
		210.089	0	0
		211.086	100	7

Table S7 Results of the reaction of PES in daylight and in the dark at different temperatures.

reaction time [min]	temperature [°C]	m/z signals	Relative Abundance [%]	
			Light	dark
60	0	181.076	-	0
		209.107	-	100
		225.102	-	2
	25	181.076	<1	<1
		209.107	27	100
		225.102	100	2

Table S8 Analysis results of PMS and PES solids. The Lot# is provided in the materials section (see above).

Phenazine species	Sample #	N[%]	C [%]	H [%]	S [%]
PMS	theoretical values	9.14	54.89	4.61	10.47
	1	8.97	54.71	4.70	10.45
	2	9.21	55.04	4.45	10.22
	3	9.32	54.05	4.41	10.58
PES	theoretical values	8.38	57.47	5.43	9.59
	1	8.25	57.25	5.49	9.77
	2	8.31	57.28	5.44	9.55
	3	8.32	57.61	5.44	9.56
	4	8.35	57.56	5.49	9.46

Table S9 Mass spectroscopy results of the storage of Wurster's Blue for 150 min and 21 d.

Reaction time	Temperature [°C]	m/z signals	Relative Abundance [%]
150 min	25	112.958	9
		144.984	15
		164.131	100
		180.125	14
21 d	25	112.958	58
		144.984	100
		164.131	6
		180.958	>1

Supporting Figures

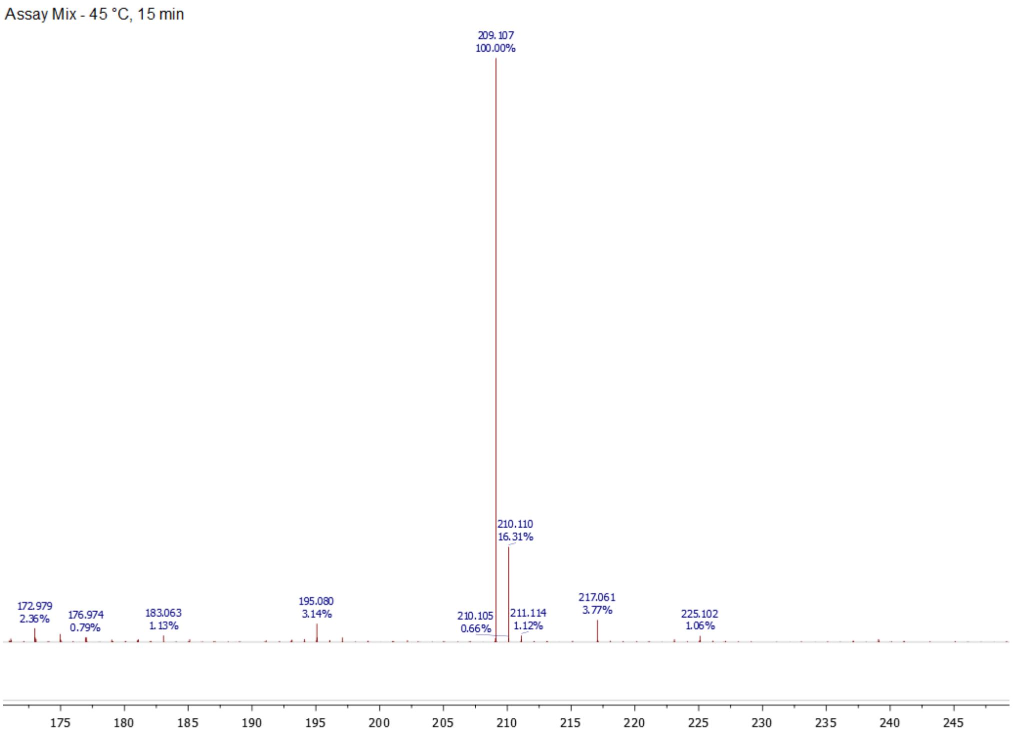

Figure S1 Mass spectrum collected of an assay mixture that had been conditioned at 45 °C for 15 min under exclusion of light.

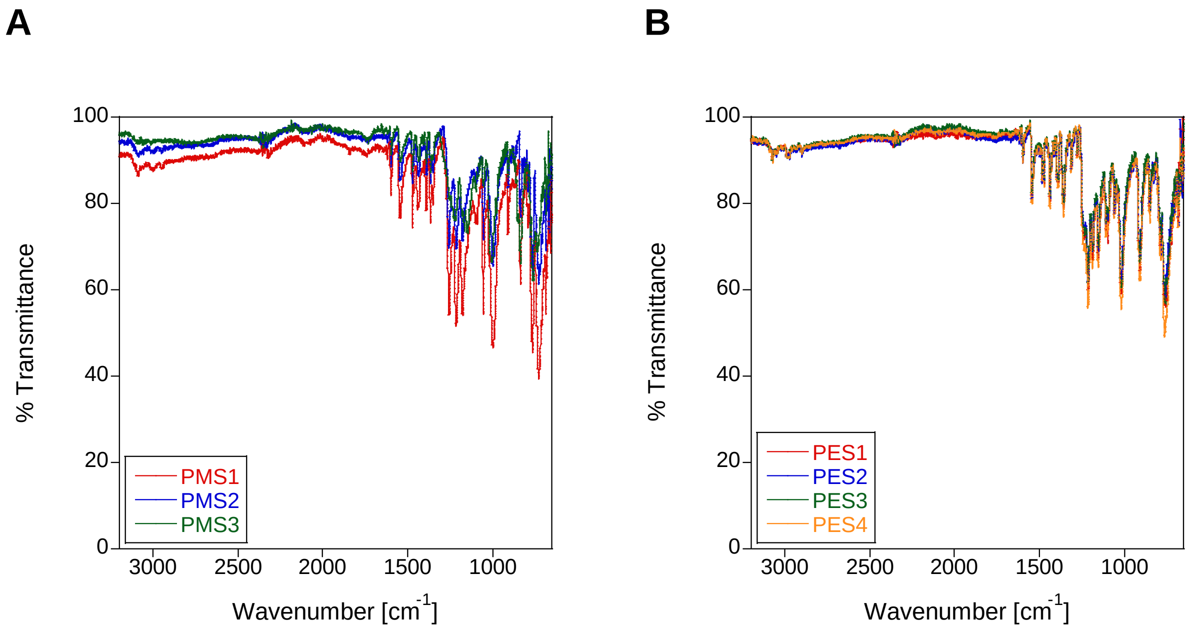

Figure S2 FTIR- spectra of different PMS (A) and PES (B) batches.

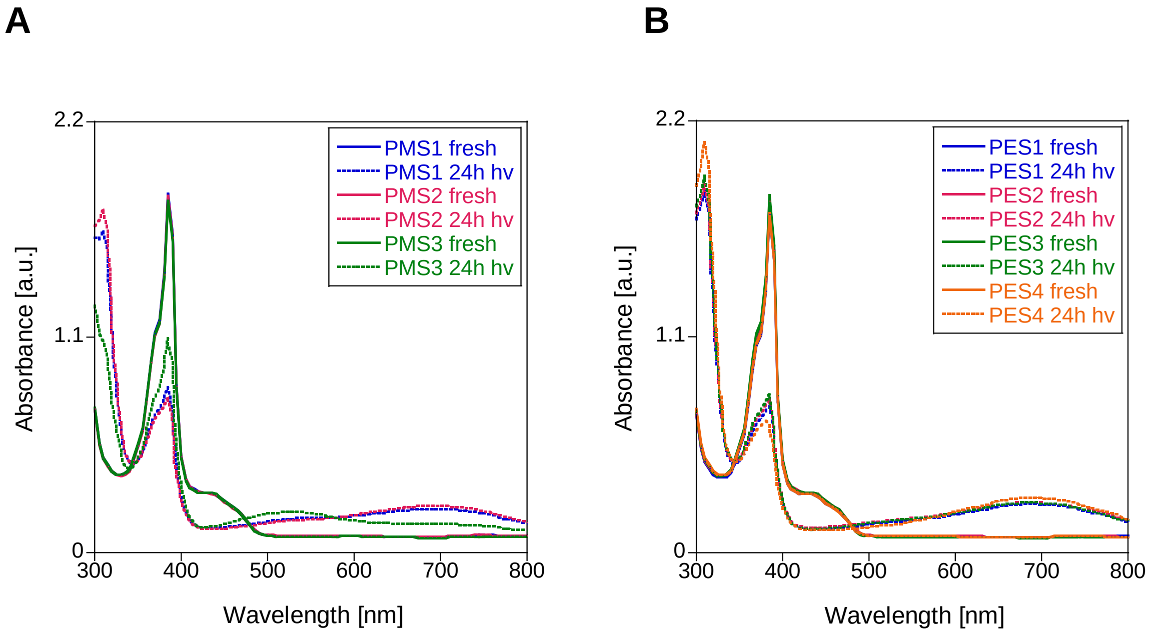

Figure S3 UV-vis spectra of different PMS (A) and PES (B) batches at RT. Conditions were as follows: 125 µM phenazine species in MilliQ before (continuous line) and after daylight exposure for 24 h (dotted line), Epoch2 plate reader (not baseline corrected).

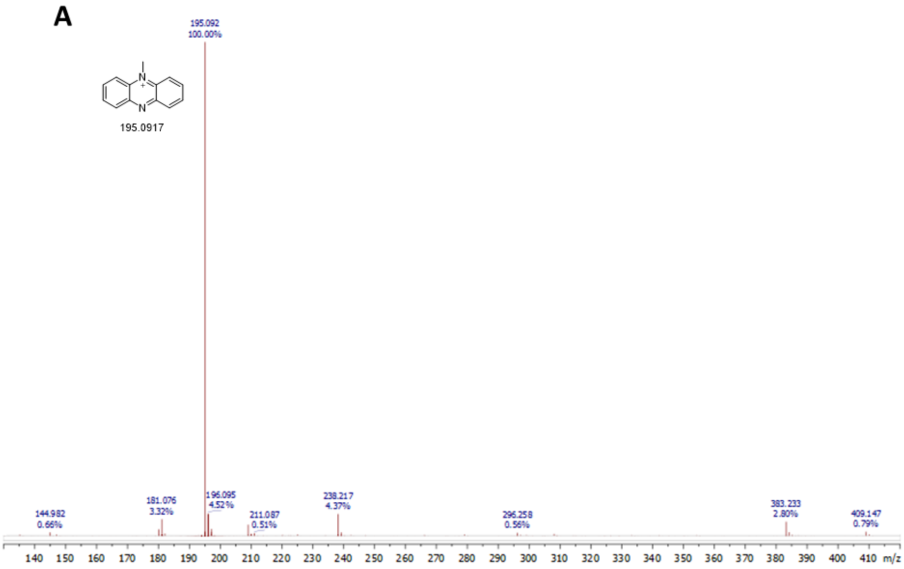

Figure S4 Mass spectrum collected of the PMS3 sample. This sample shows besides the signals seen in PMS1 and PMS2 samples (181.076 m/z, 195.092 m/z and 196.095 m/z) additional fragments.

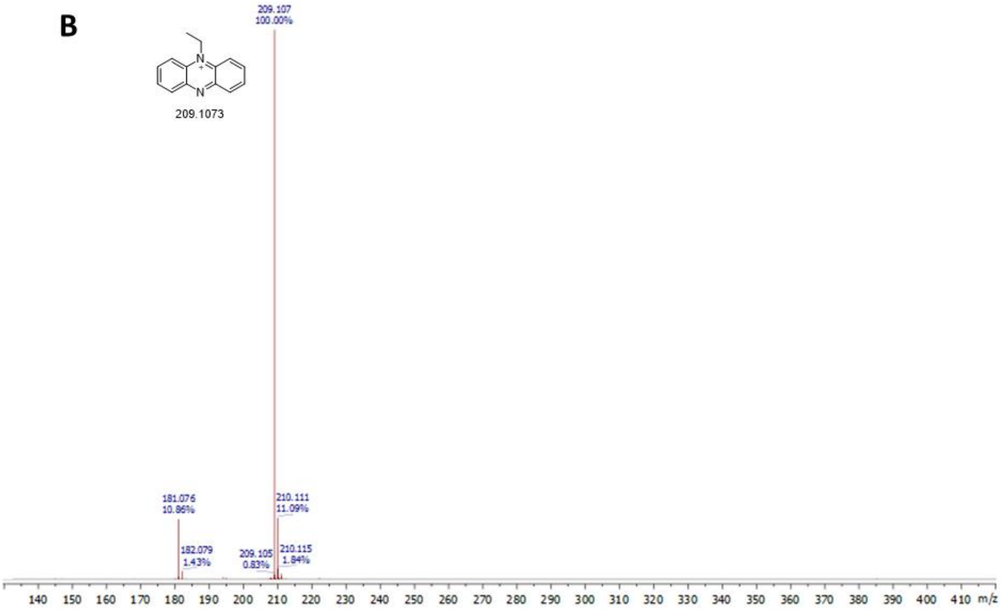

Figure S5 Mass spectrum collected of the PES4 sample as example for all PES samples exhibiting a similar fragmentation.

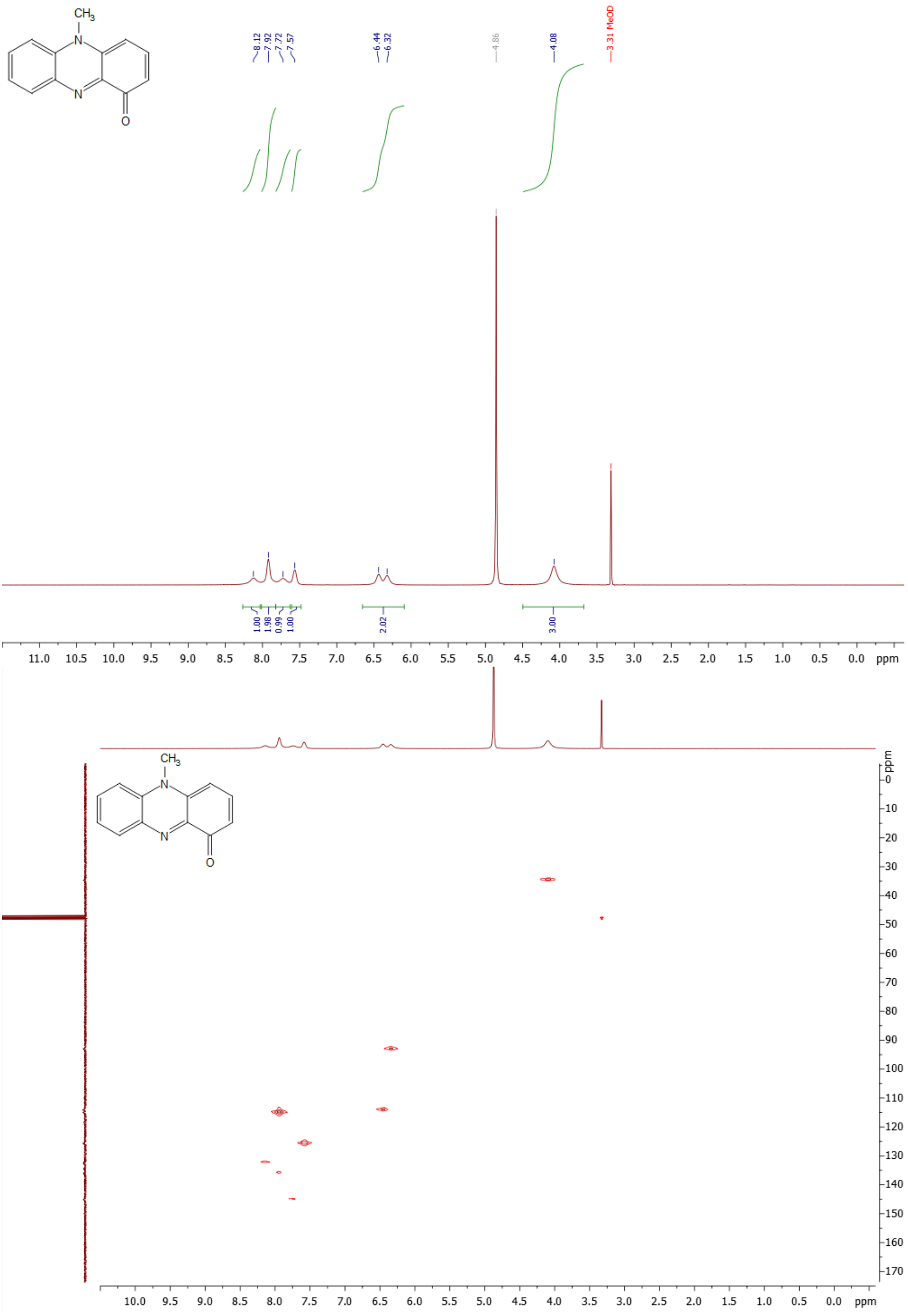

Figure S6 NMR spectra of pyocyanin.

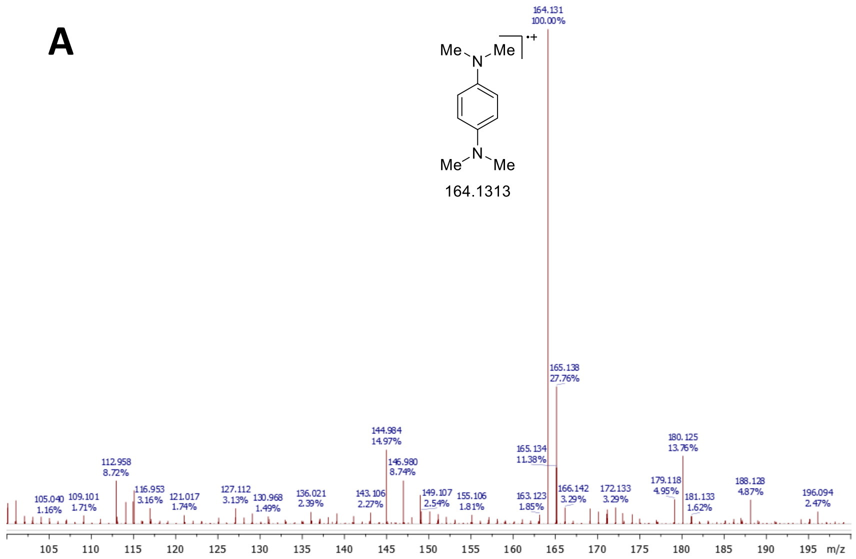

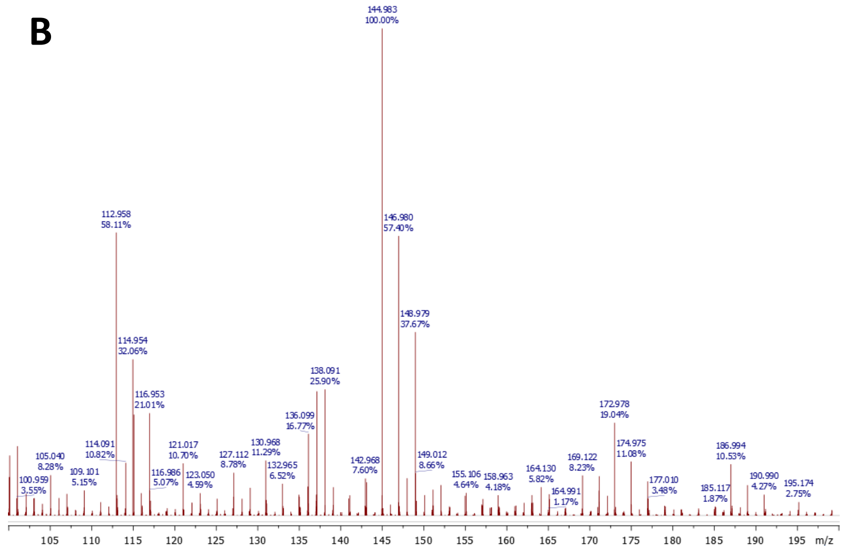

Figure S7 ESI-MS spectra of a (A) sample of WB that was stored for 150 min and (B) a sample that was stored for 21 days at room temperature in amber tubes.

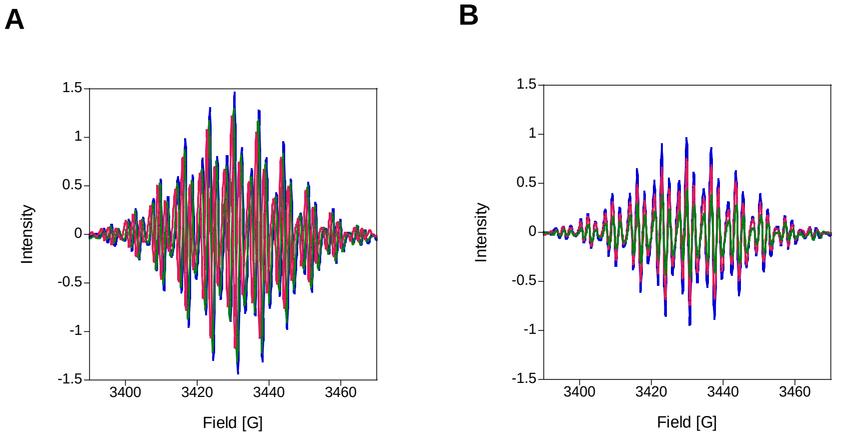

Figure S8 EPR spectra of 200 µM WB in 100 mM multicomponent buffer. Blue line: fresh sample, pink line: heated sample for 30 min at 30 °C, green line: heated sample for 30 min at 45 °C both under exclusion of light. (A) pH 7.2 (B) pH 9. Spectra were collected on an EMXnano EPR spectrometer at room temperature and in the dark.

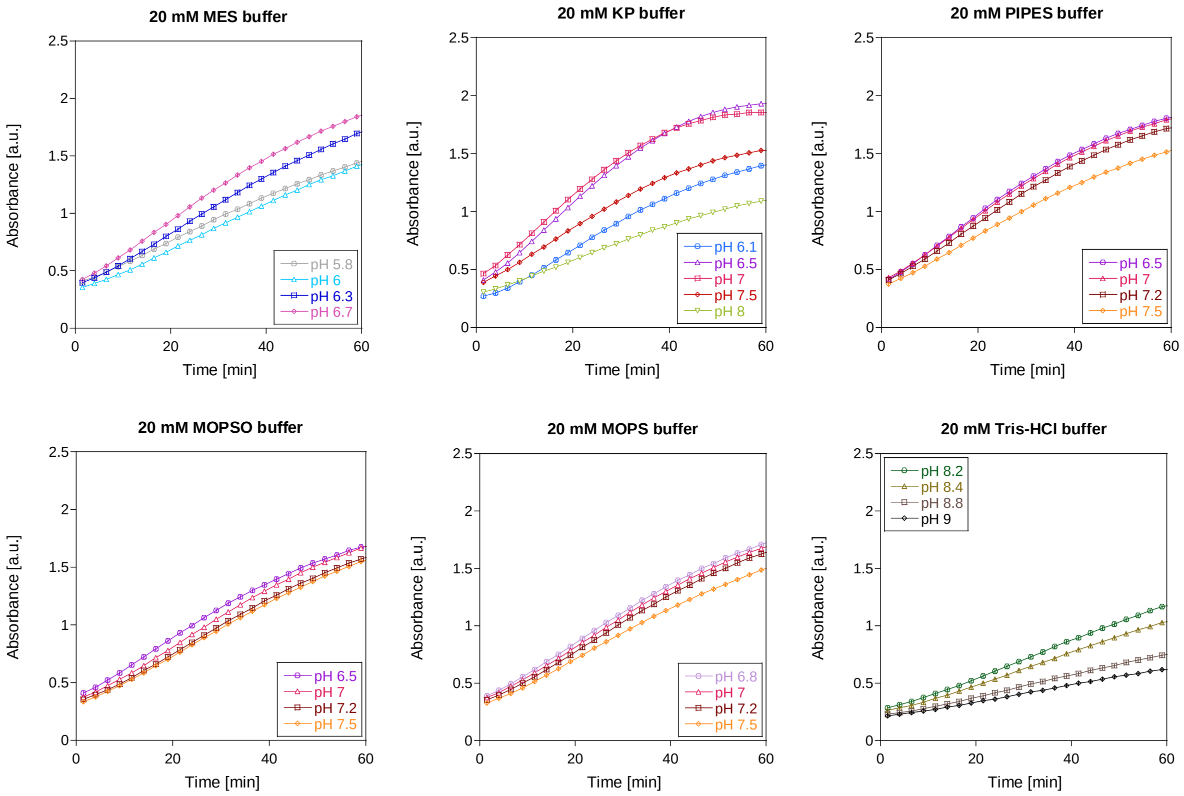

Figure S9 pH-dependence of in situ WB formation from N,N,N′,N′-Tetramethyl-p-phenylenediamine (TMPD) in different buffers. Conditions were as follows: 200 µM TMPD in 20 mM buffer of different pH, heated for 1 h at 45 °C. Absorbance at 610 nm was monitored with an Epoch2 plate reader. Triplicates with standard deviation are shown. Data were path length corrected.

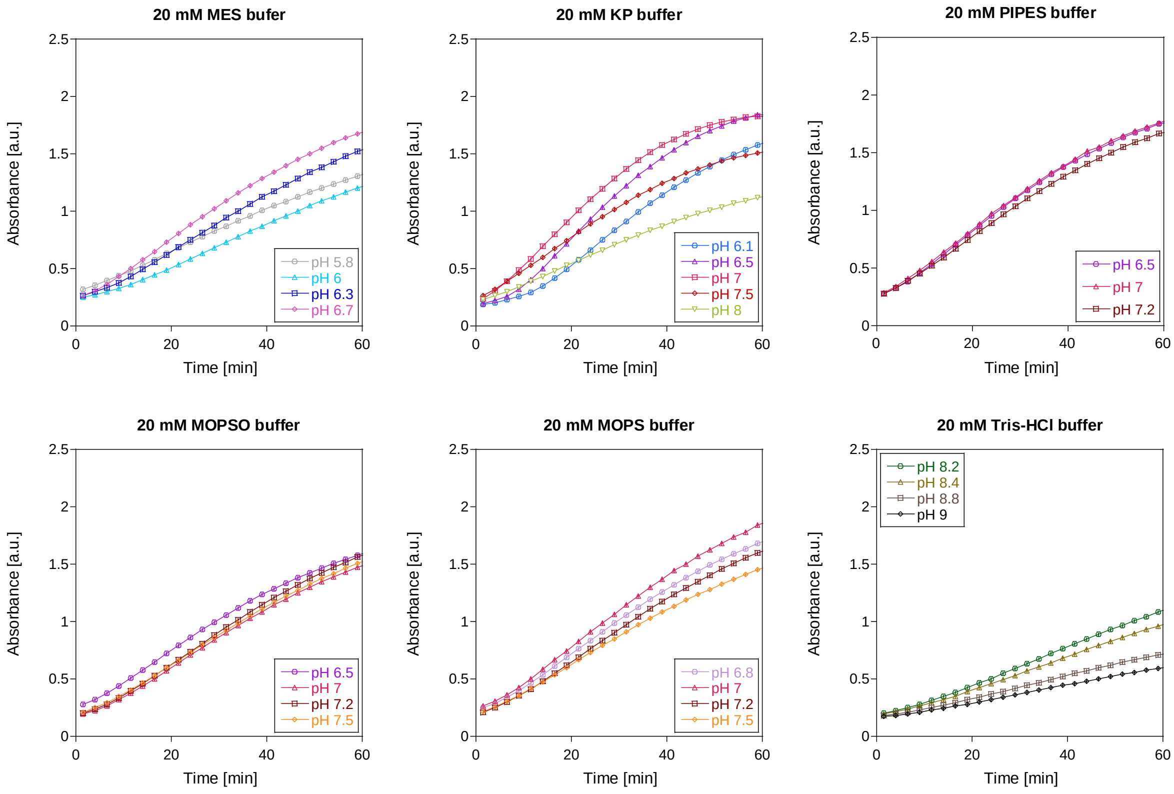

Figure S10 pH-dependence and *in situ* WB formation from N,N,N′,N′-Tetramethyl-p-phenylenediamine dihydrochloride (TMPDD) in different buffers. Conditions were as follows: 200 µM TMPDD in 20 mM buffer of different pH, heated for 1 h at 45 °C. Absorbance at 610 nm was monitored with an Epoch2 plate reader. Triplicates with standard deviation are shown. Data were path length corrected.

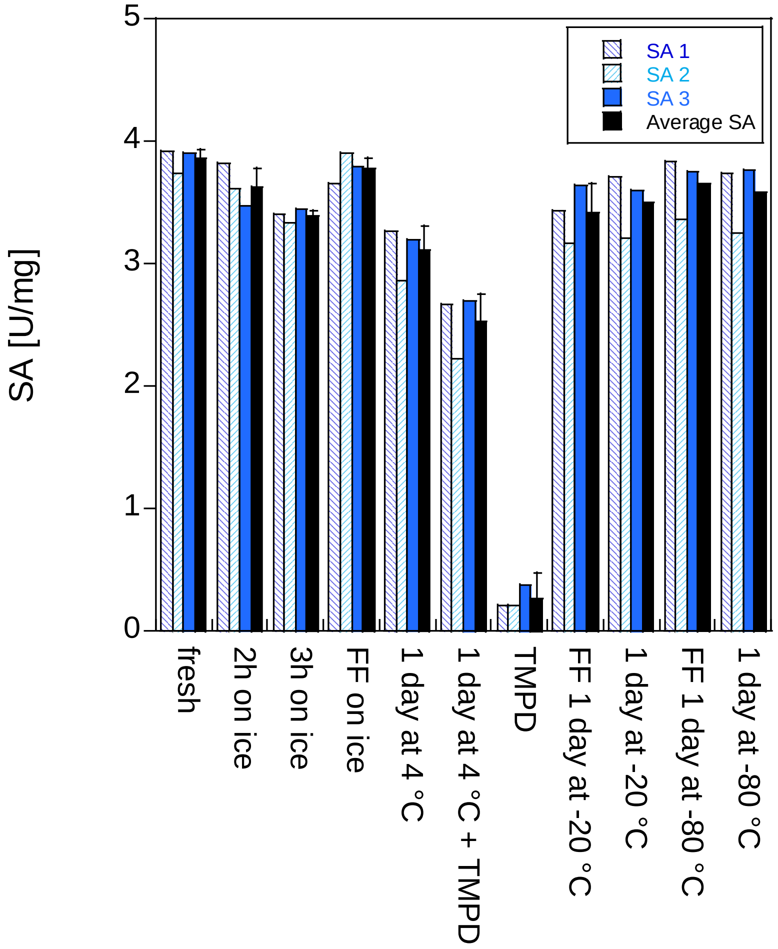

Figure S11 Influence of WB storage conditions on the specific activity (SA, in µmol min^{-1} mg^{-1}) of His-tagged AM1 La-MDH. WB samples were either stored on ice over a prolonged time or under different conditions with and without flash freezing (FF) the samples. The assay conditions were as follows: 100 mM MC buffer pH 9, 100 nM AM1 La-MDH, 200 µM WB, 15 mM NH_4Cl, 50 mM MeOH. Volume in wells 200 µL. The WB concentration 200 µM for each condition. Data were collected using an Epoch2 plate reader with automatic path length correction to 1 cm at 30 °C and 610 nm. Technical duplicates were conducted by the same pair of hands. Initial rates (first 30 s of the assay) were used for calculating the SA.

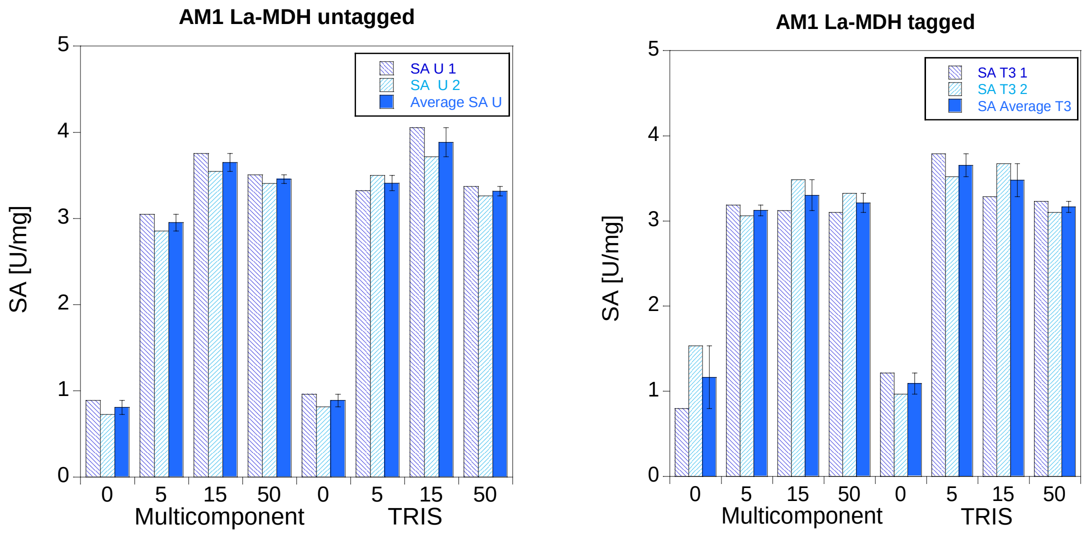

Figure S12 Ammonia-dependence of AM1 La-MDH in 100 mM multicomponent and 25 mM TRIS buffer pH 9. The specific activity (SA, in µmol min^{-1} mg^{-1}) of untagged (U) and His-tagged (T3) La-MDH was determined using WB. The conditions were as follows: 200 µM WB and increasing concentrations of NH_4Cl (0, 5, 15 and 50 mM), 50 mM MeOH. The total volume in wells was 222 µL. Data were collected at 30 °C and 610 nm using an Epoch2 plate reader. All SA are technical replicates. SA1 and SA2 were determined by different pairs of hands.

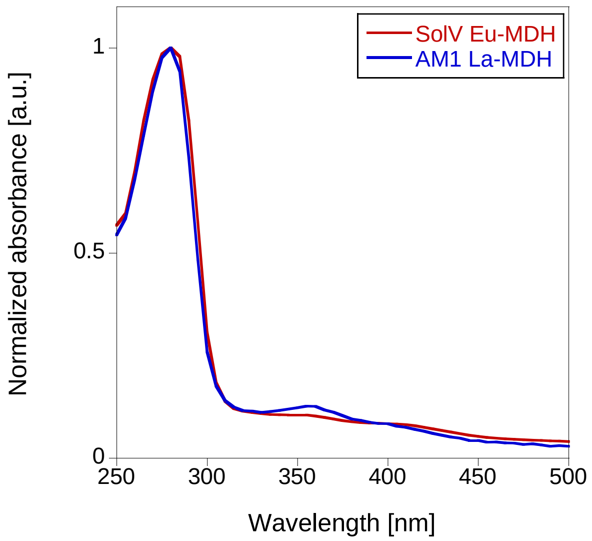

Figure S13 Normalized absorbance spectra of SolV Eu-MDH (red) vs. AM1 La-MDH untagged (blue) both in 20 mM PIPES pH 7.2 and after washing. Spectra were recorded at room temperature at a Cary60 spectrophotometer. Here the PQQ to total protein ratios (355/289) were 0.10486 for Eu-MDH and 0.12629 for La-MDH.

References

1 L. Michaelis and S. Granick (1943) J Am Chem Soc 65:1747-1755
2 H. McIlwain (1937) J. Chem. Soc., 1937, 1704-1711
3 C. Anthony (2000) Subcellular Biochemistry 35:73-117
4 F. Kehrmann and E. Havas (1913) Ber Dtsch Chem Ges 46:341-352
5 B. Jahn, A. Pol, H. Lumpe, T. Barends, A. Dietl, C. Hogendoorn, H. Op den Camp and L. Daumann (2018) ChemBioChem 19:1147-1153
